# Supplementary figures and images for: Comprehensive Analysis of Pyroptosis-Associated in Molecular Classification, Immunity and Prognostic of Glioma
Source: Front Genet. 2022 Jan 7;12:781538. doi: 10.3389/fgene.2021.781538 (PMC8777075; doi:10.3389/fgene.2021.781538)

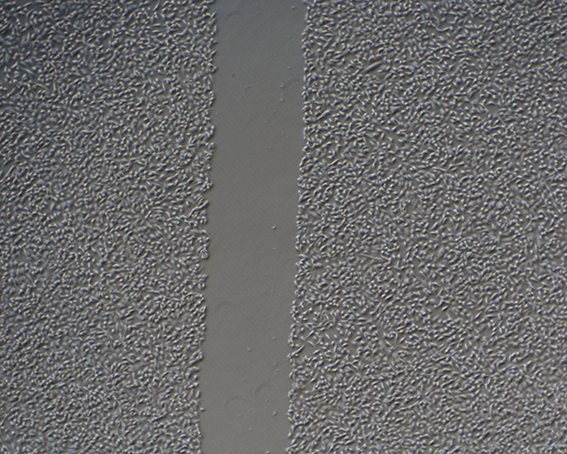

Supplement: Supplementary file 1 [file DataSheet3.ZIP › Cell scratchy assay/LN229-CASP8si-0h.tif]

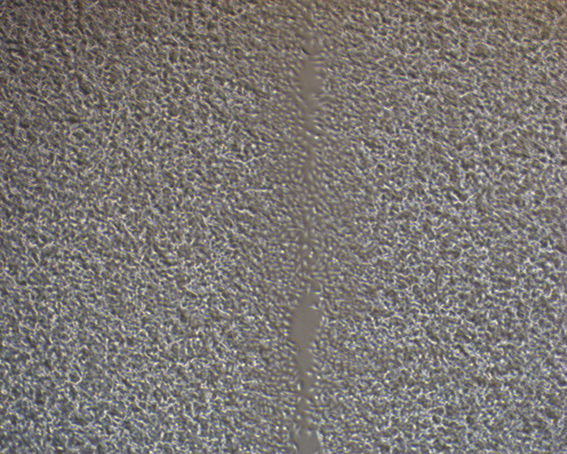

Supplement: Supplementary file 1 [file DataSheet3.ZIP › Cell scratchy assay/LN229-CASP8si-48h.tif]

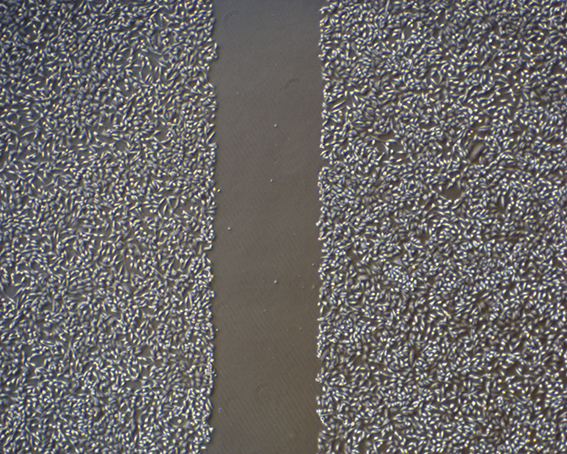

Supplement: Supplementary file 1 [file DataSheet3.ZIP › Cell scratchy assay/LN229-NC-0h.tif]

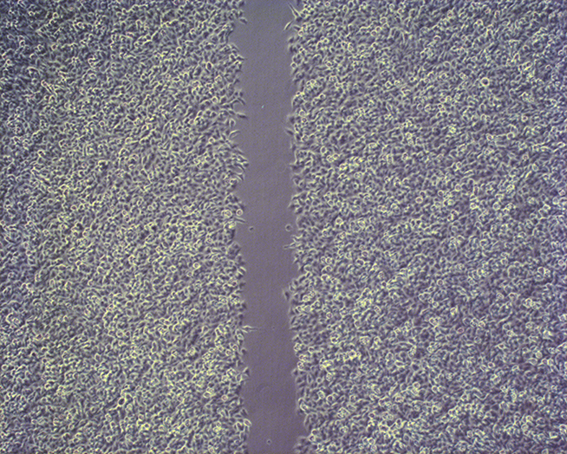

Supplement: Supplementary file 1 [file DataSheet3.ZIP › Cell scratchy assay/LN229-NC-48h.tif]

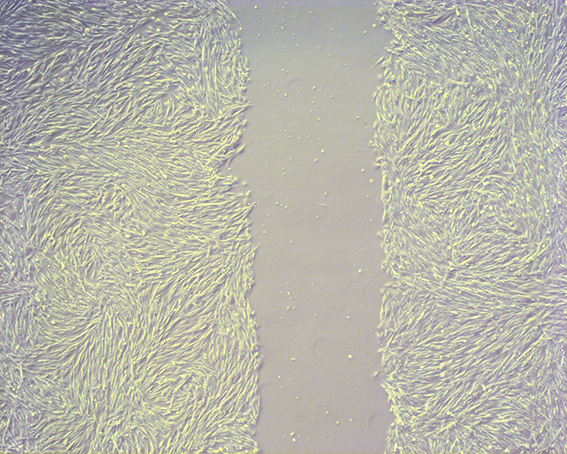

Supplement: Supplementary file 1 [file DataSheet3.ZIP › Cell scratchy assay/U87-CASP8si-0h.tif]

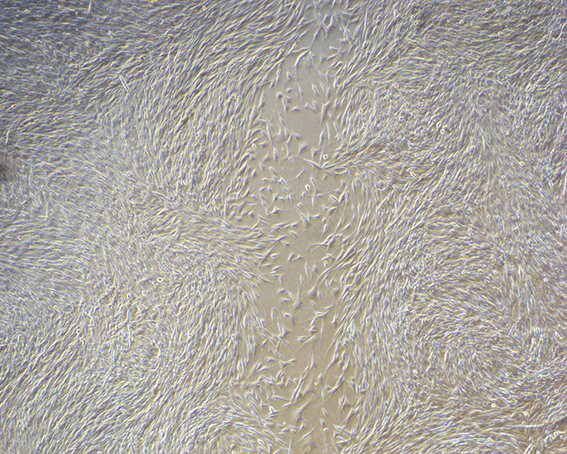

Supplement: Supplementary file 1 [file DataSheet3.ZIP › Cell scratchy assay/U87-CASP8si-48h.tif]

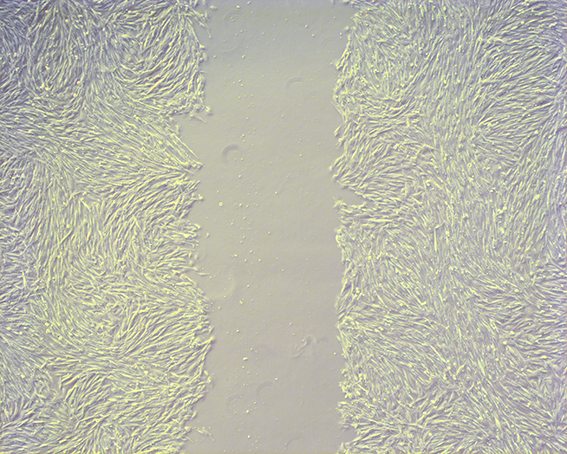

Supplement: Supplementary file 1 [file DataSheet3.ZIP › Cell scratchy assay/U87-NC-0h.tif]

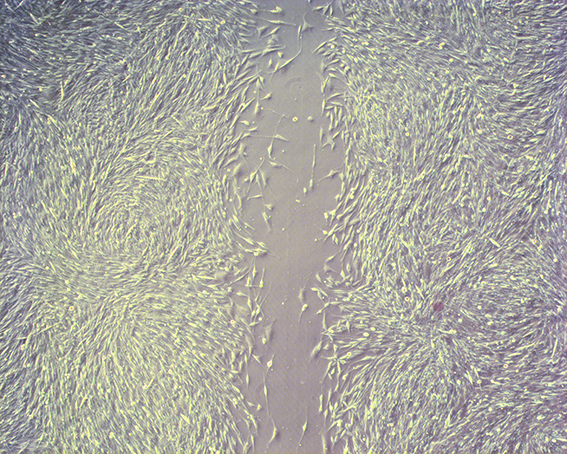

Supplement: Supplementary file 1 [file DataSheet3.ZIP › Cell scratchy assay/U87-NC-48h.tif]

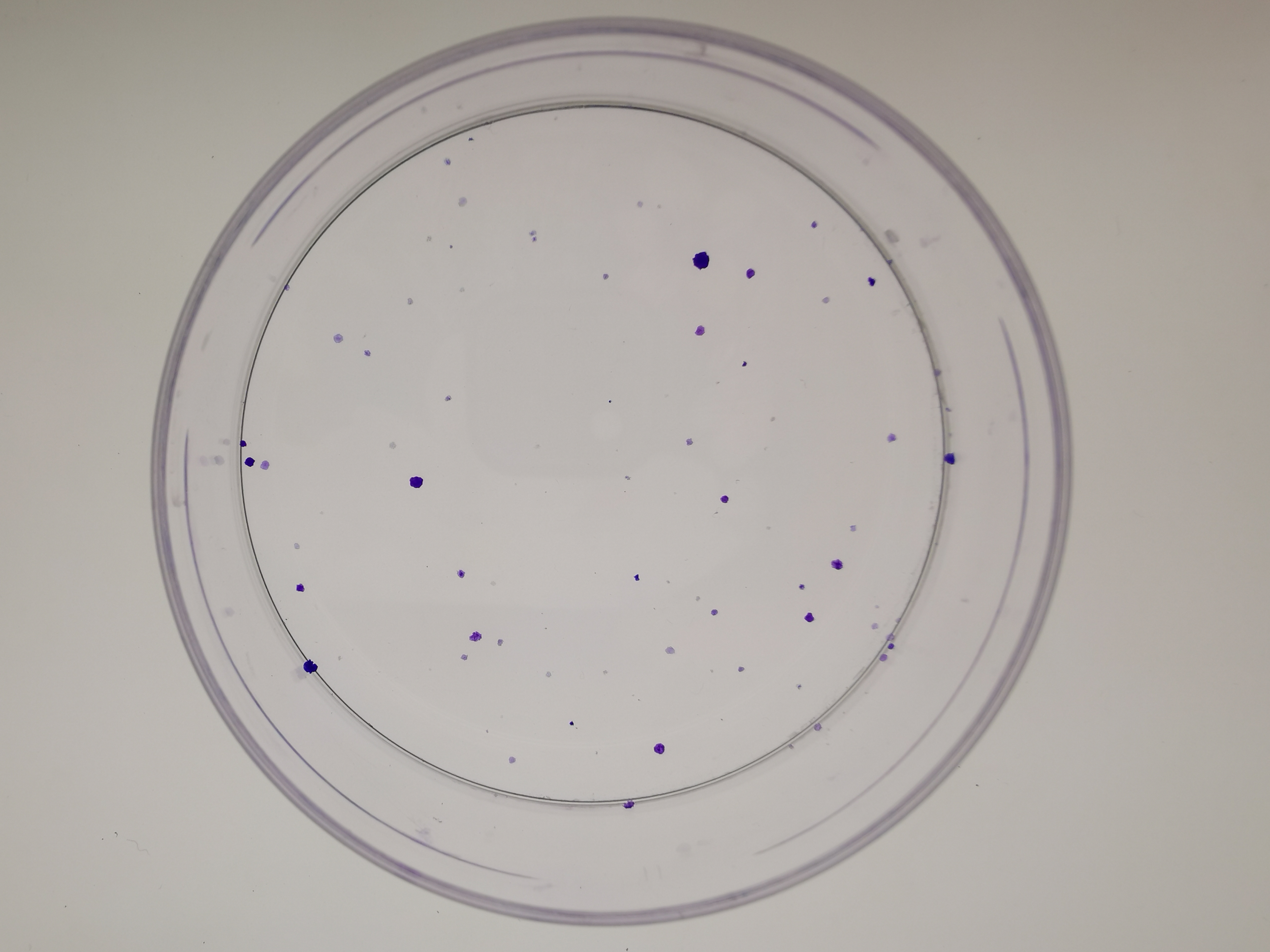

Supplement: Supplementary file 3 [file DataSheet4.ZIP › Clonogenic assay/LN229-CASP8si.jpg]

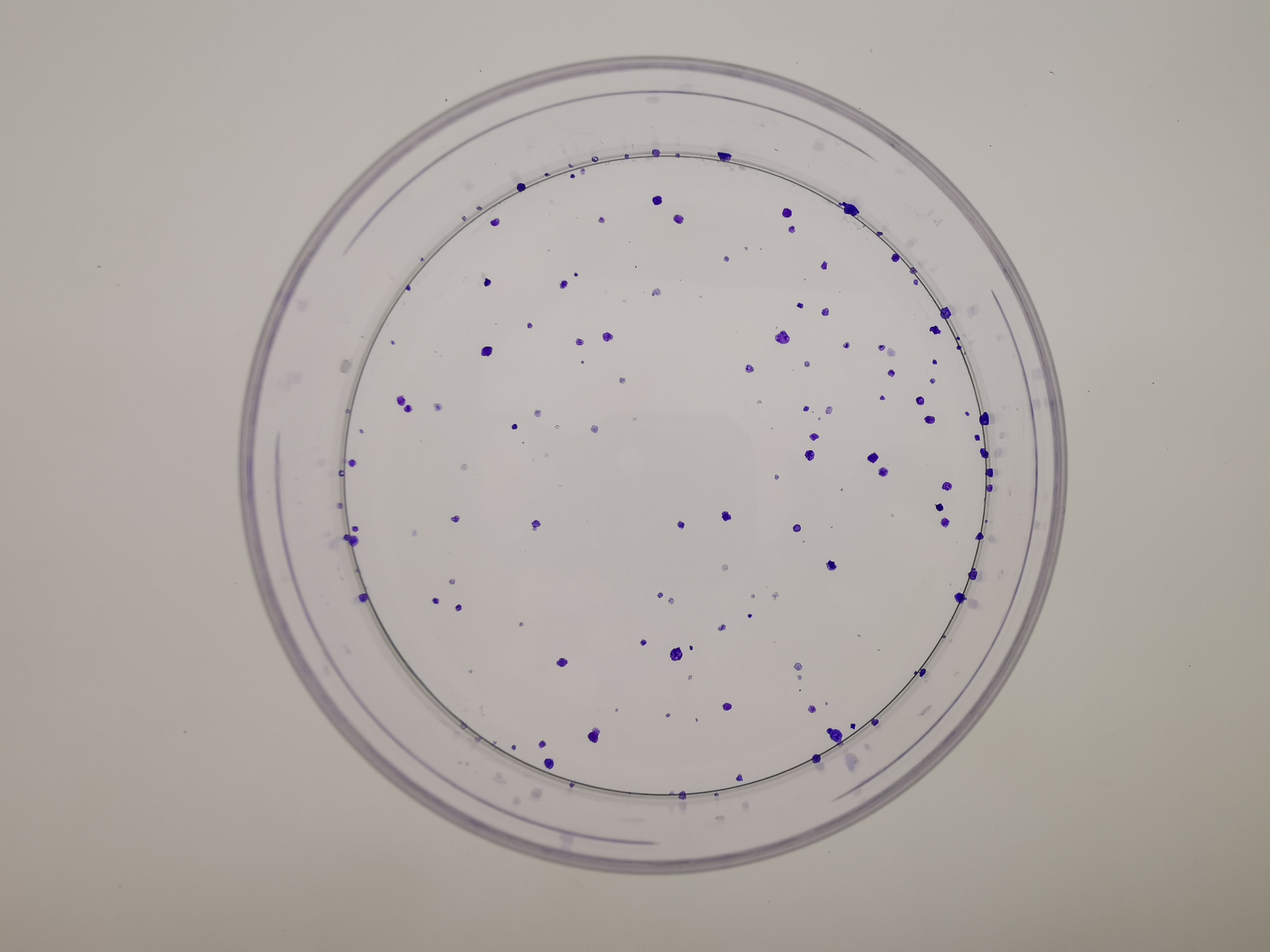

Supplement: Supplementary file 3 [file DataSheet4.ZIP › Clonogenic assay/LN229-NC.jpg]

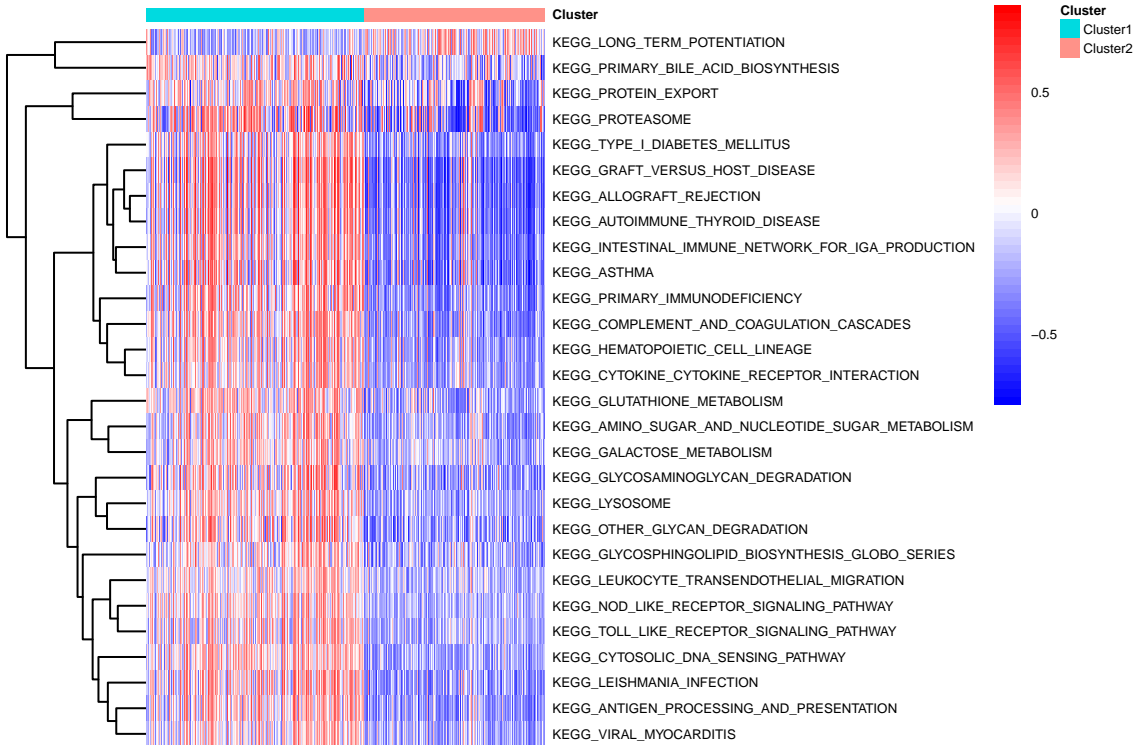

Supplement: Supplementary file 5 [file DataSheet6.ZIP › Additional file 4/01.GSVA/heatmap.pdf]

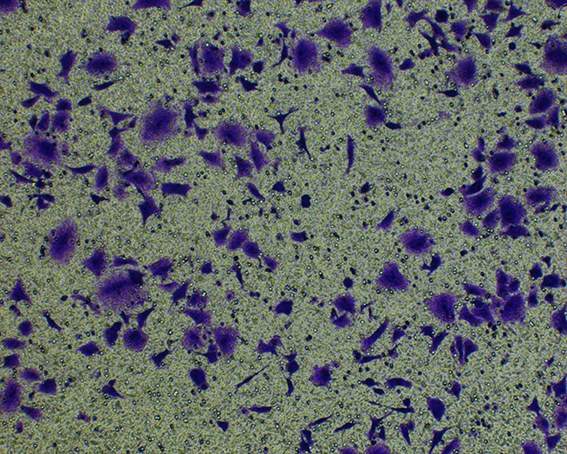


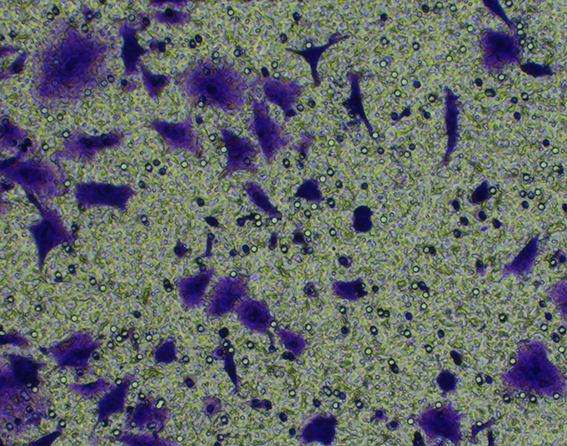

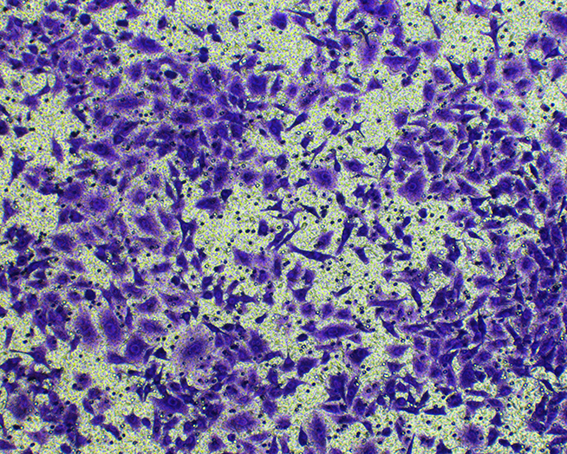

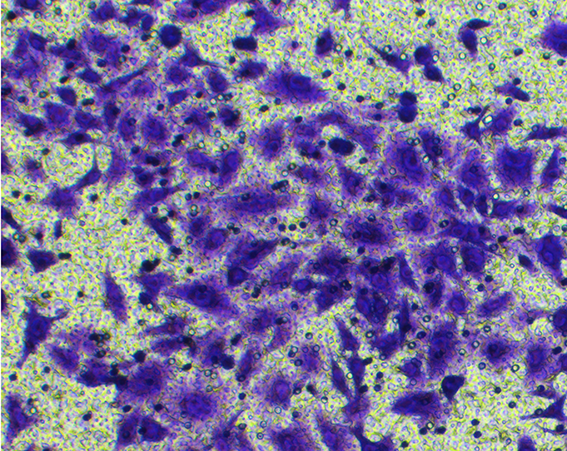

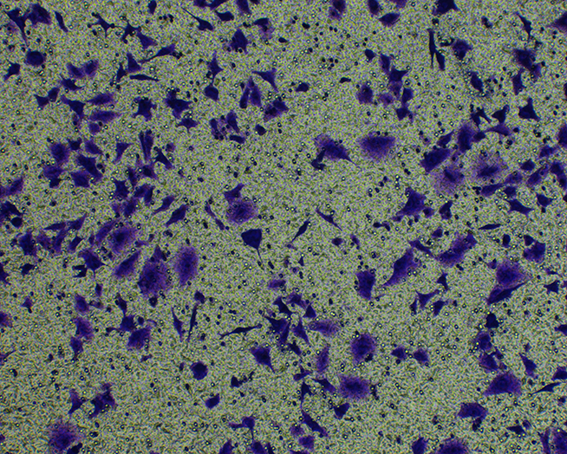

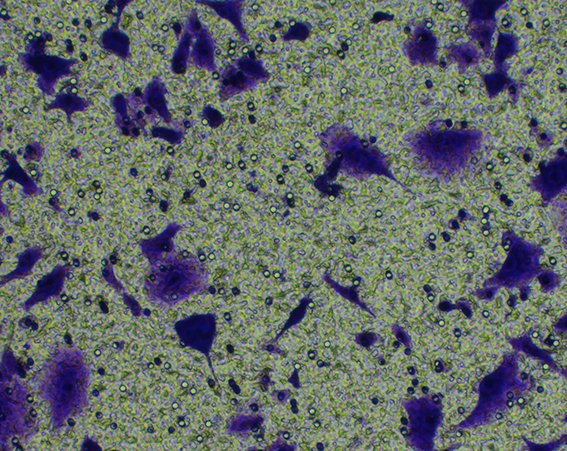

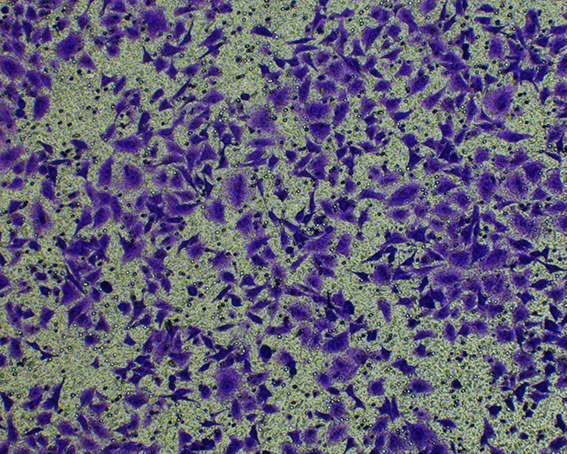

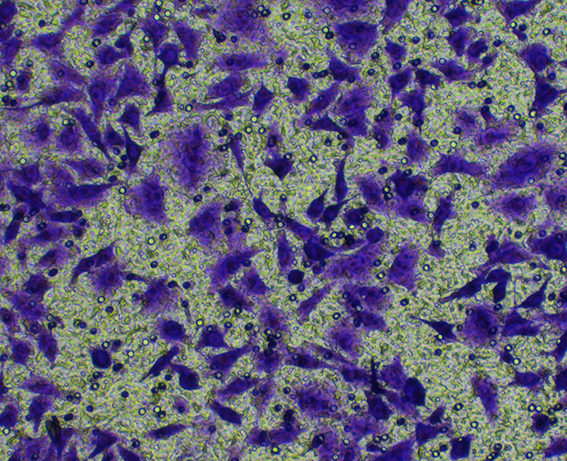

Supplement: Supplementary file 7 [file DataSheet2.ZIP › Cell migration assay/Cell migration assay.docx]

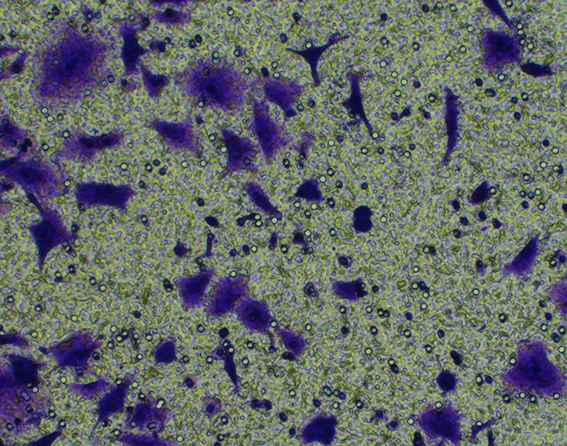

Supplement: Supplementary file 7 [file DataSheet2.ZIP › Cell migration assay/LN229-CASP8si-10X.tif]

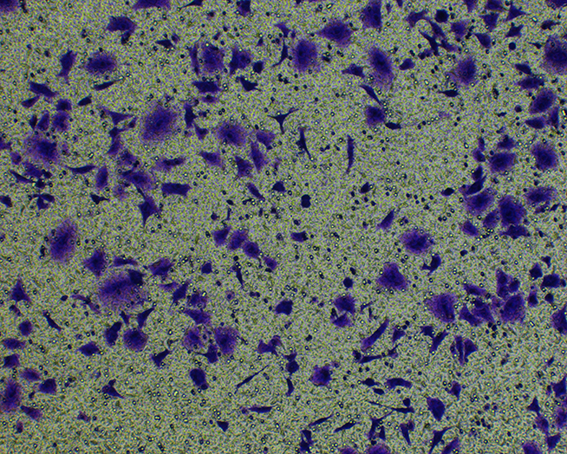

Supplement: Supplementary file 7 [file DataSheet2.ZIP › Cell migration assay/LN229-CASP8si-5X.tif]

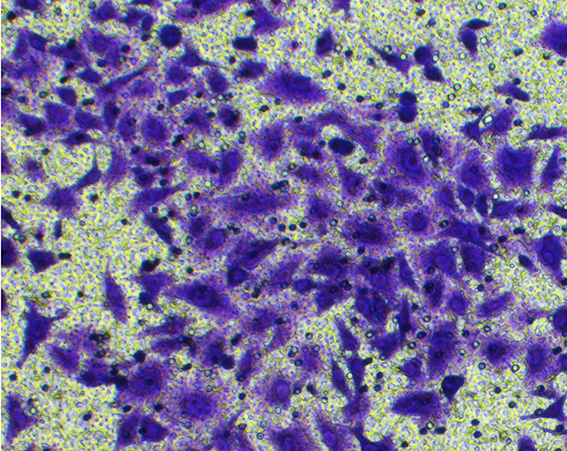

Supplement: Supplementary file 7 [file DataSheet2.ZIP › Cell migration assay/LN229-NC-10X.tif]

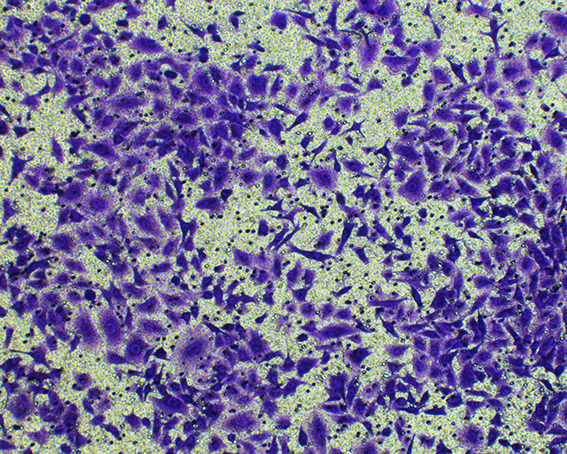

Supplement: Supplementary file 7 [file DataSheet2.ZIP › Cell migration assay/LN229-NC-5X.tif]

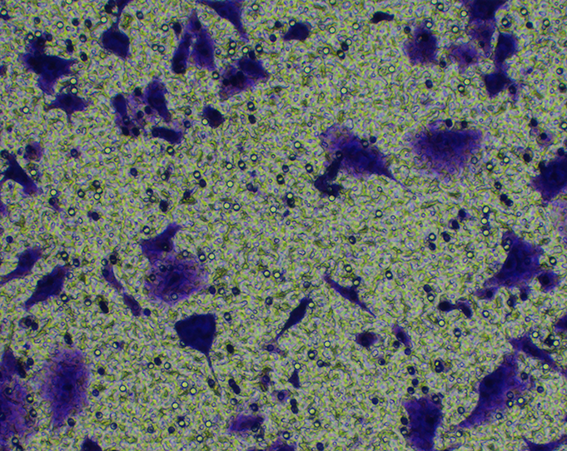

Supplement: Supplementary file 7 [file DataSheet2.ZIP › Cell migration assay/U87-CASP8si-10X.tif]

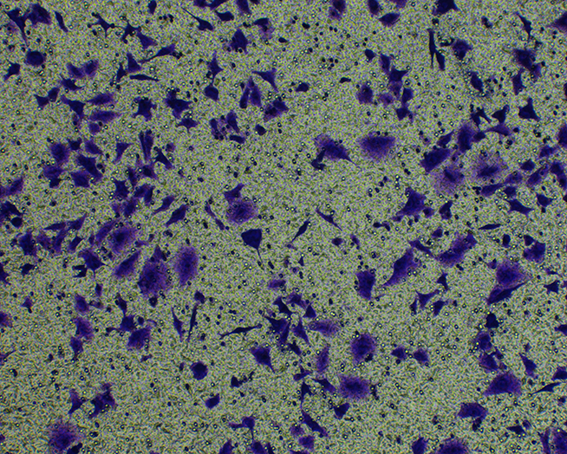

Supplement: Supplementary file 7 [file DataSheet2.ZIP › Cell migration assay/U87-CASP8si-5X.tif]

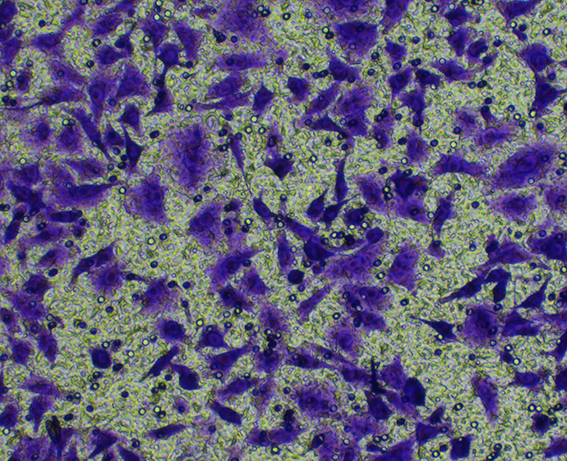

Supplement: Supplementary file 7 [file DataSheet2.ZIP › Cell migration assay/U87-NC-10X.tif]

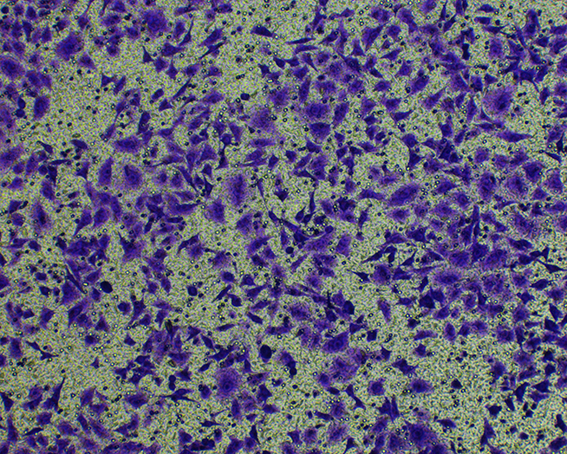

Supplement: Supplementary file 7 [file DataSheet2.ZIP › Cell migration assay/U87-NC-5X.tif]

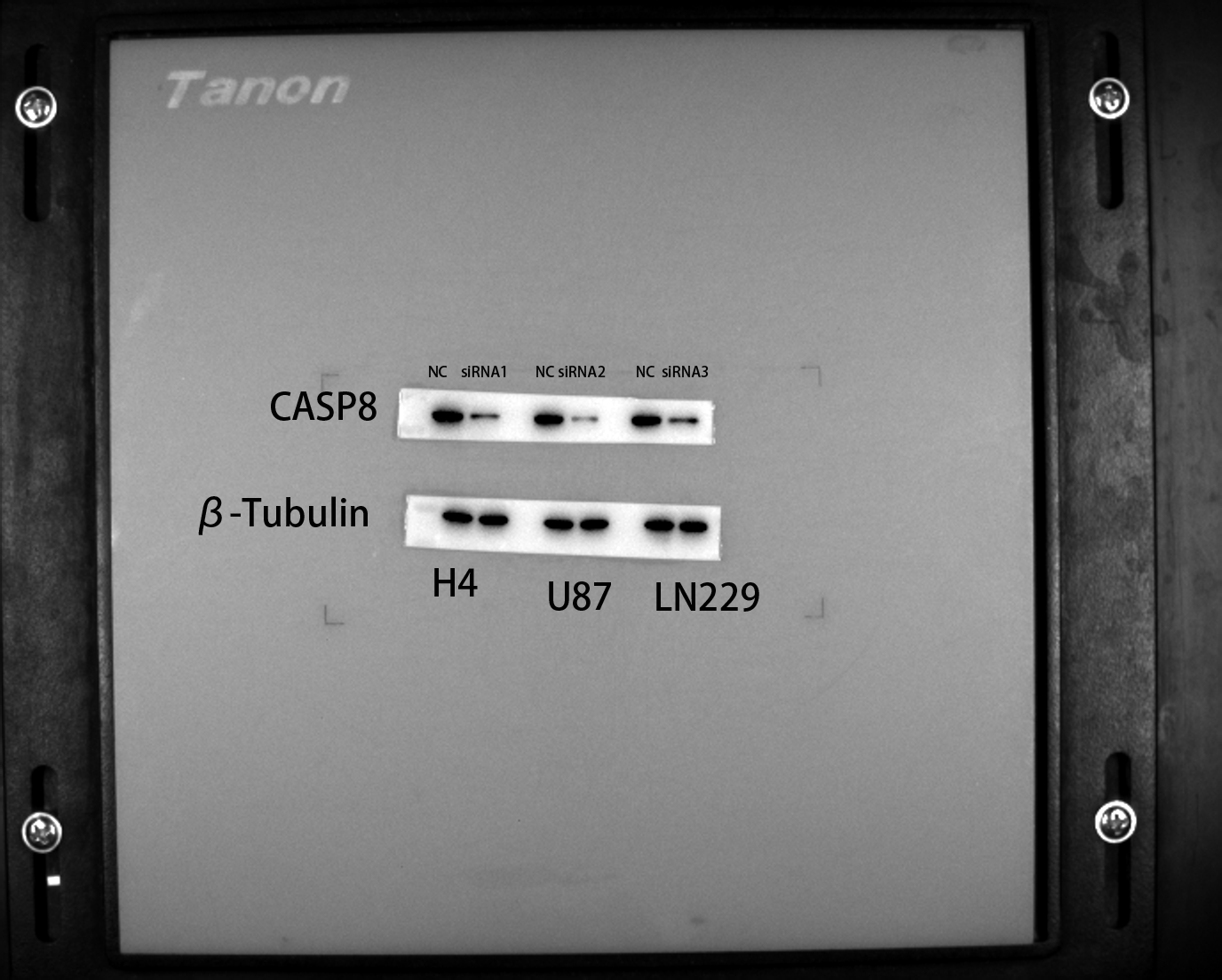

Supplement: Supplementary file 9 [file DataSheet5.ZIP › Western_blot/Figure 10B-1.Tif]

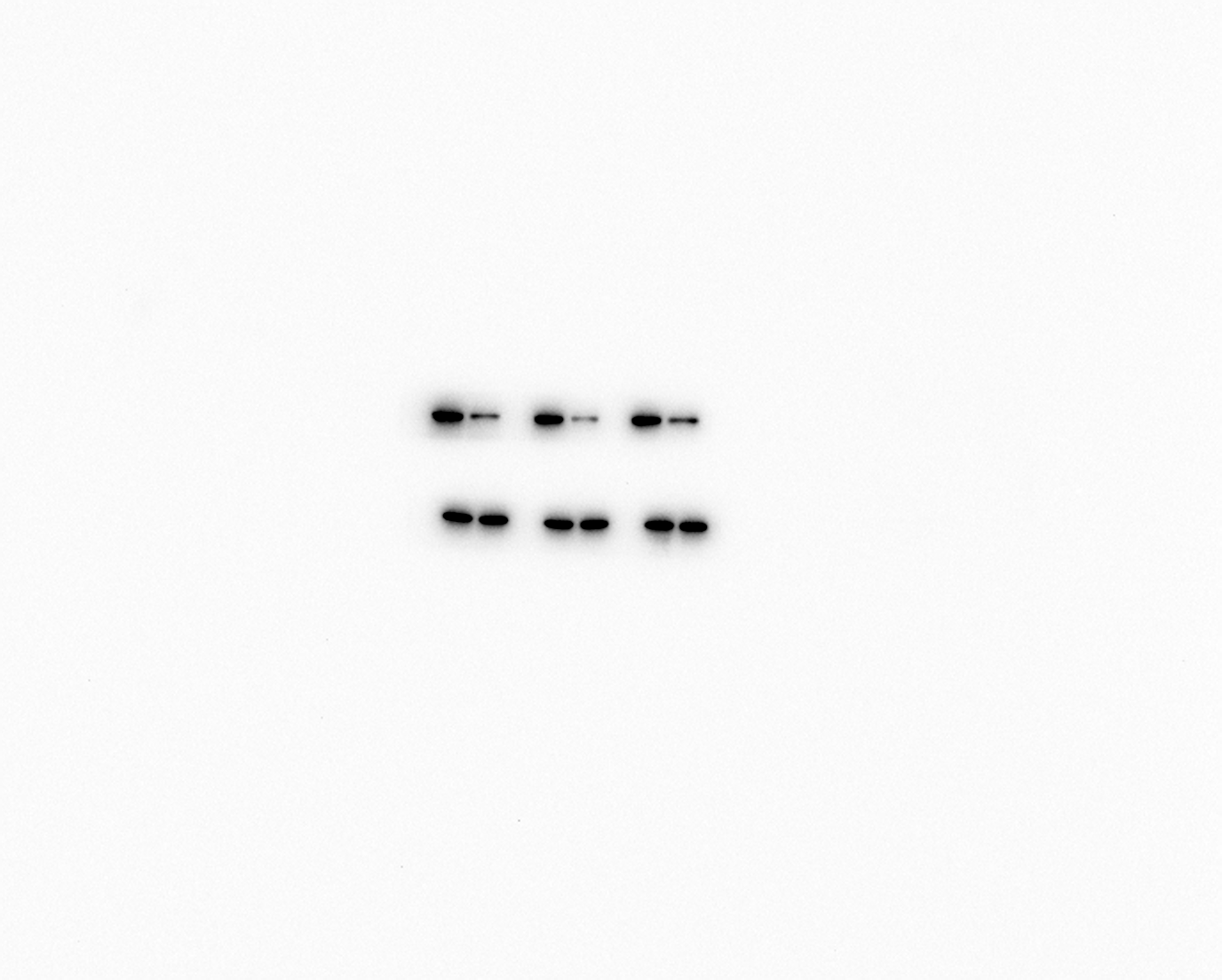

Supplement: Supplementary file 9 [file DataSheet5.ZIP › Western_blot/Figure 10B-2.Tif]

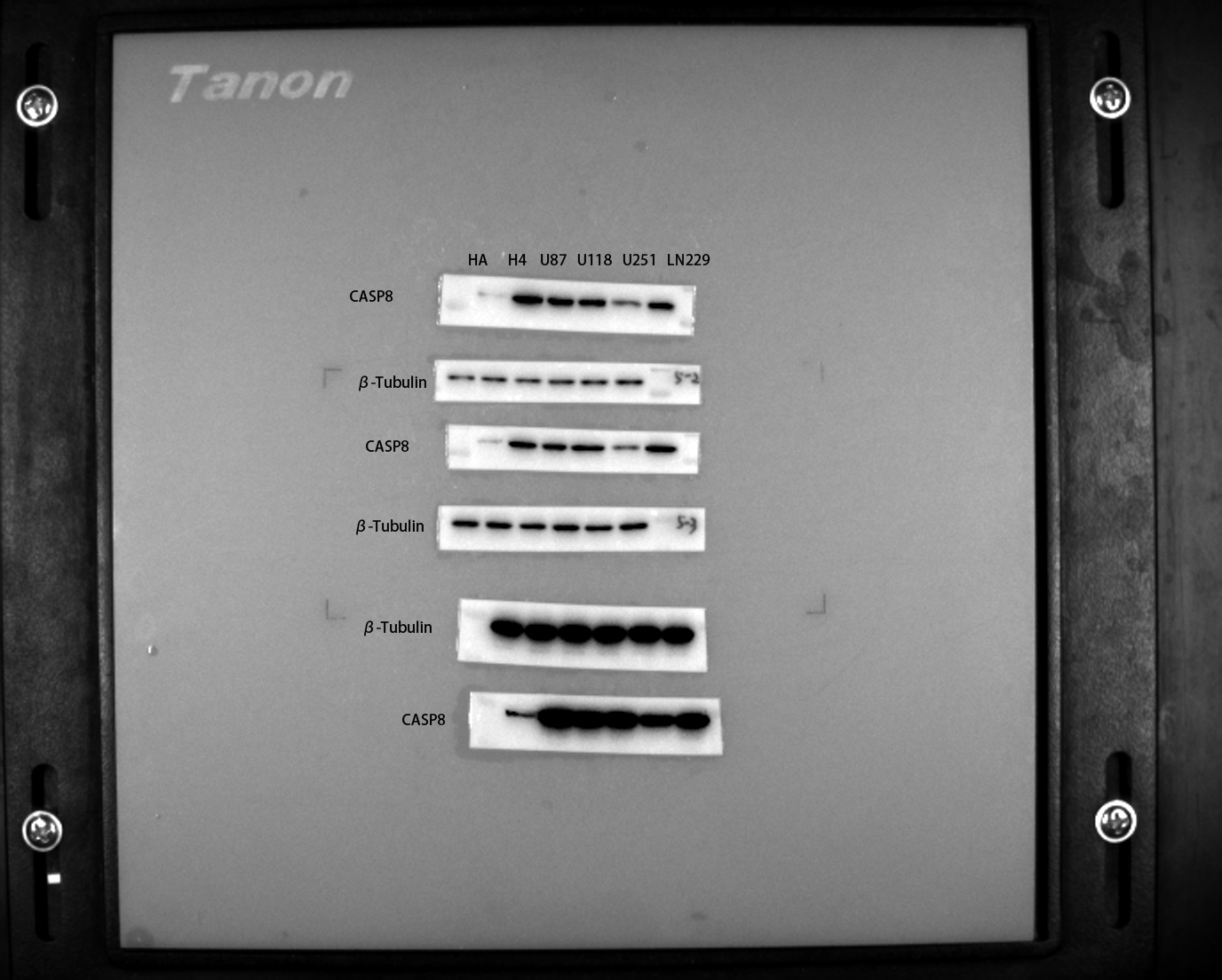

Supplement: Supplementary file 9 [file DataSheet5.ZIP › Western_blot/Figure-1.tif]

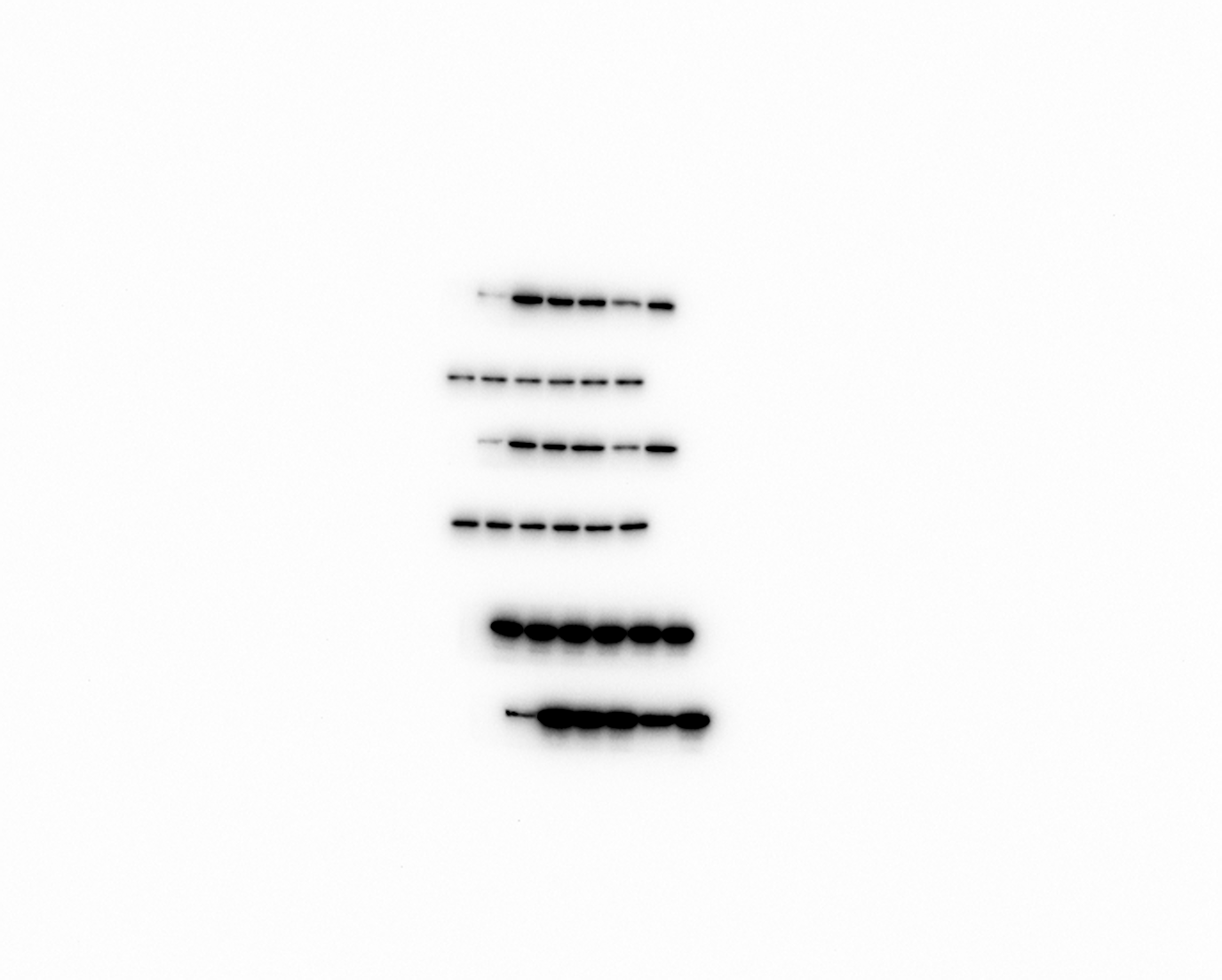

Supplement: Supplementary file 9 [file DataSheet5.ZIP › Western_blot/Figure10A-2.Tif]
