# Supplementary material for: Comprehensive Analysis of Pyroptosis-Associated in Molecular Classification, Immunity and Prognostic of Glioma
Source: Front Genet. 2022 Jan 7;12:781538. doi: 10.3389/fgene.2021.781538 (PMC8777075; doi:10.3389/fgene.2021.781538)
Supplement: Supplementary file 2 [file DataSheet7.docx]

**Supplementary material 1** The details of experiments process in vitro

**Methods for experiments in vitro**

**Cell lines and Western blot**

The human astrocytes cell line was purchased from BIONEED (Beijing, China), and U87, H4, U251, U118 and LN229 were purchased from BeNa Culture Collection (Beijing, China). All cells were cultured in DMEM (Sigma, St. Louis, MO, USA) containing 10% fetal bovine serum at 37 °C with 5% CO2. The CASP8 small interference RNA (siRNA) and negative control (NC) were synthesized by Santa-cruz Biotechnology Co, Inc. All cells were transfected with 40 nM siRNA or vectors for 24 hours. We extracted proteins from cells using RIPA buffer. The BCA Kit was adopted to detect the protein concentrations. The membranes were incubated with rabbit anti-human primary antibodies against CASP8 (ab32397, 1:5000 dilution, Abcam, USA), or β-Tubulin (ab8226, 1:10000 dilution, Abcam) as a loading control.

**Quantitative polymerase chain reaction**

Total RNA was isolated using TRIzol (Invitrogen). 1,000ng RNA was reverse-transcribed into cDNA in a total reaction volume of 20Δl with PrimeScriptTM RT Reagent Kit according to manufacturer’s instructions (Takara). The qPCR primer sequences were designed by Santa-cruz Biotechnology Co, Inc. Real-time PCR was performed using SYBR premix Taq via CFX96 Real-Time PCR Detection System (Bio-Rad, Richmond, CA, USA). The 2^-ΔΔCT^ method was used to calculate the relative expression of CASP8.

**Cell migration assay**

The cell migration assay was performed using 24-well transwell chambers with 600 μl DMEM and 10%FBS at the bottom. The NC cell and glioma (U87 and LN229) cells (1x 10^5^) with transfection of CASP8 siRNA were seeded into upper chambers with 100 ul serum free medium. After 24 hours of culture, the cells on the upper surface of the filter were removed with cotton swabs. The invading cells on the lower surface were fixed with 4% formaldehyde, stained with Giemsa solution.

**Cell scratchy assay**

We firstly draw a horizontal line behind 6-well plate using marker, about every 0.5-1cm, across the hole. Approximately 1x10^5^ NC siRNA, U87 and LN229 siRNA cells were seeded into in the 6-well plate. After 48 hours, the cell monolayer was scraped with a sterile 200-μL pipette tip. Then we wash the cells with PBS for three times for removing the extracted cells, and added serum free medium. All cells were incubated in 37°C with 5% CO_2_. Photos were taken at 0 and 48 hours.

**Clonogenic assay**

NC siRNA, U87 and LN229 siRNA cells were seeded in 6-well plates at densities of 1 × 10^5^ per well. after 2 weeks, cells were washed three times with cold PBS, and then stained with 0.2% crystal violet (containing methanol). Colonies consisting of more than 50 cells were defined as surviving colonies. All viability measurements are normalized with the NC siRNA group.
