# Supplementary material for: Comprehensive Analysis of Pyroptosis-Associated in Molecular Classification, Immunity and Prognostic of Glioma
Source: Front Genet. 2022 Jan 7;12:781538. doi: 10.3389/fgene.2021.781538 (PMC8777075; doi:10.3389/fgene.2021.781538)
Supplement: Supplementary file 6 [file DataSheet1.PDF]

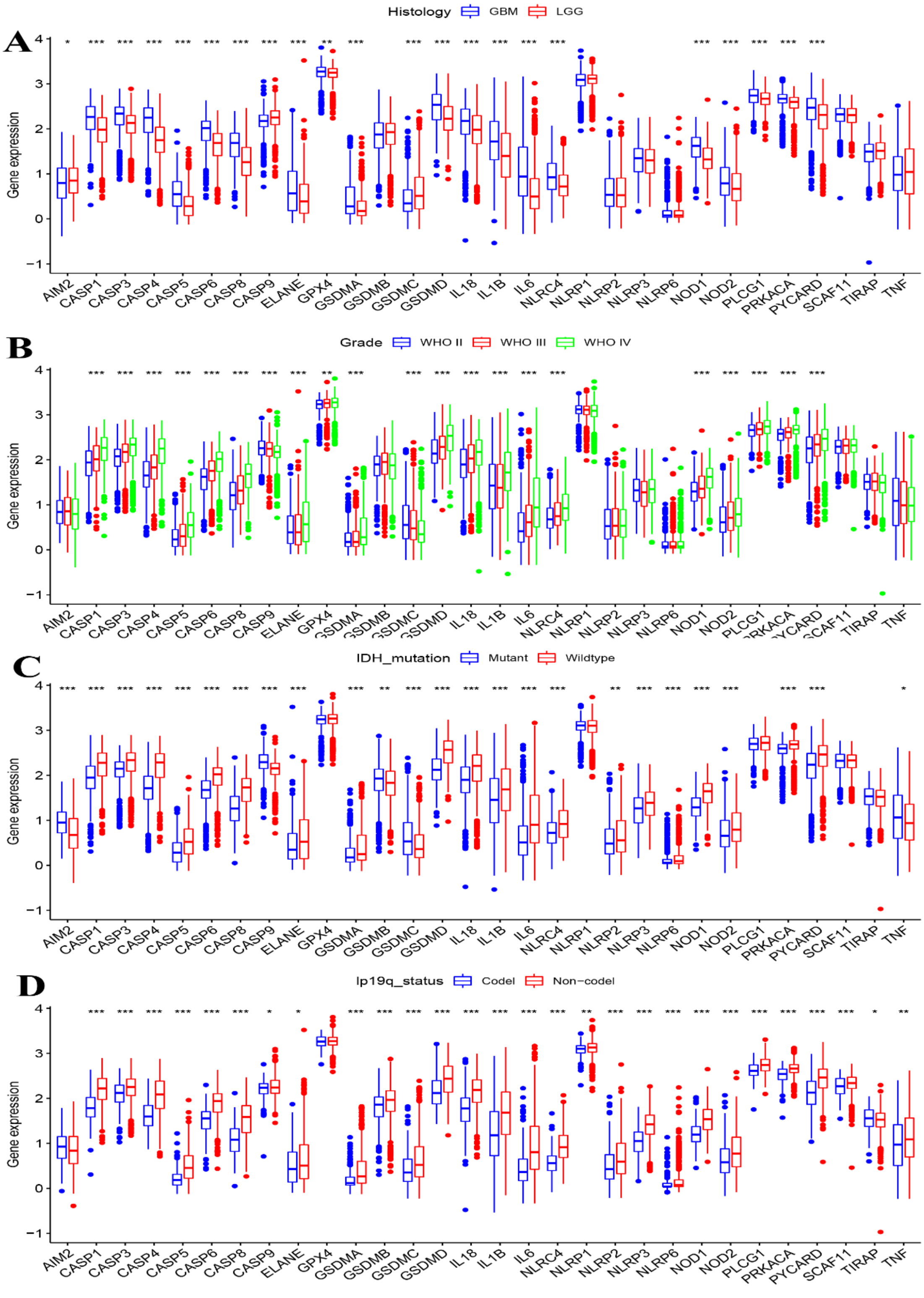
A
Histology GBM LGG
B
Grade WHO II WHO III WHO IV
C
IDH_mutation Mutant Wildtype
D
lp19q_status Codel Non-codel
Gene expression
AIM2
CASP1
CASP3
CASP4
CASP5
CASP6
CASP8
CASP9
ELANE
GPX4
GSDMA
GSDMB
GSDMC
GSDMD
IL18
IL1B
IL6
NLRC4
NLRP1
NLRP2
NLRP3
NLRP6
NOD1
NOD2
PLCG1
PRKACA
PYCARD
SCAF11
TIRAP
TNF

Figure S1

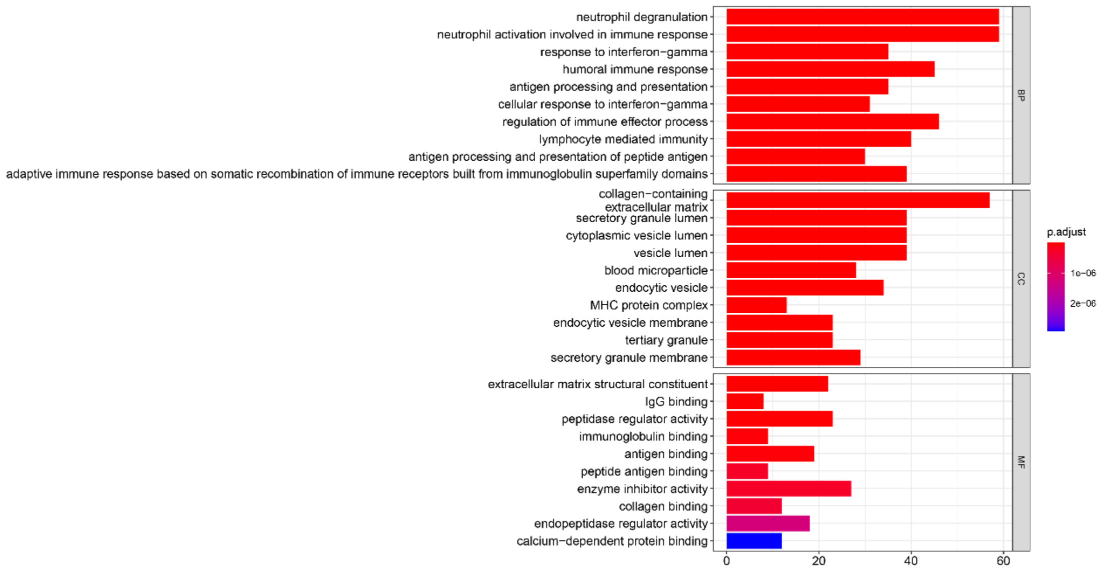

Figure S2

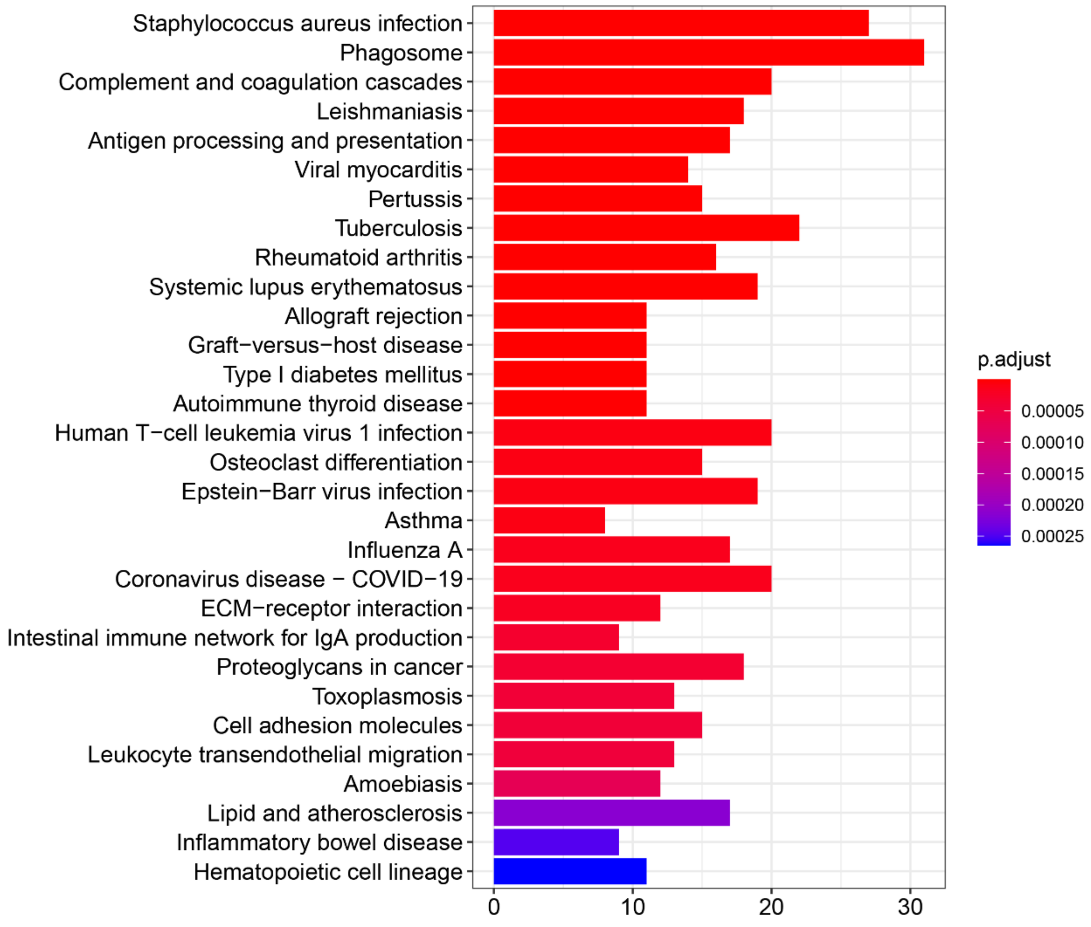

Figure S3

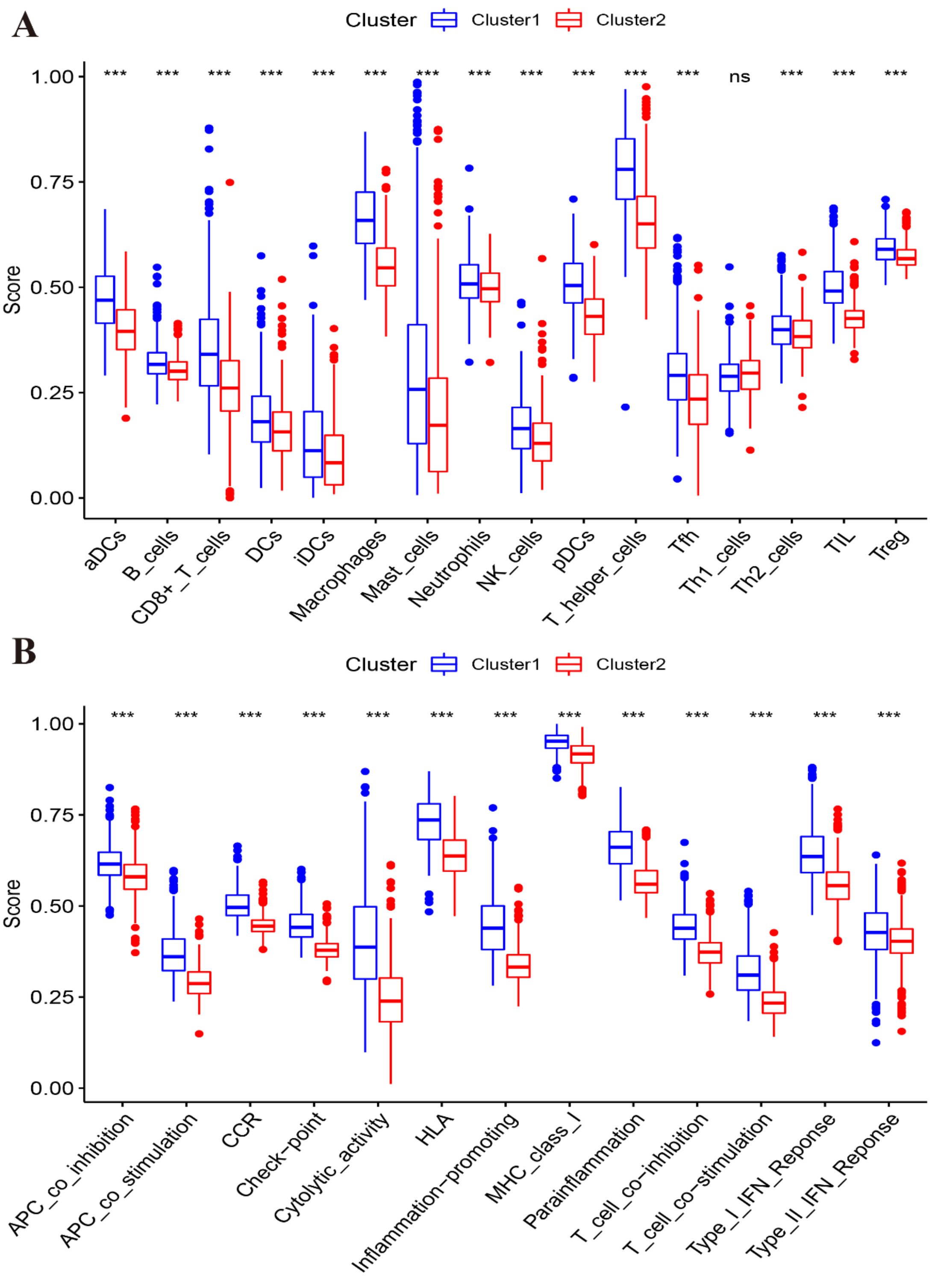
A
Cluster
Cluster1
Cluster2
Score
1.00
0.75
0.50
0.25
0.00
*** *** *** *** *** *** *** *** *** *** *** *** ns *** *** ***
aDCs
B_cells
CD8+_T_cells
DCs
iDCs
Macrophages
Mast_cells
Neutrophils
NK_cells
pDCs
T_helper_cells
Tfh
Th1_cells
Th2_cells
TIL
Treg
B
Cluster
Cluster1
Cluster2
Score
1.00
0.75
0.50
0.25
0.00
*** *** *** *** *** *** *** *** *** *** *** *** ***
APC_co_inhibition
APC_co_stimulation
CCR
Check-point
Cytolytic_activity
HLA
Inflammation-promoting
MHC_class_I
Parainflammation
T_cell_co-inhibition
T_cell_co-stimulation
Type_I_IFN_Reponse
Type_II_IFN_Reponse

Figure S4

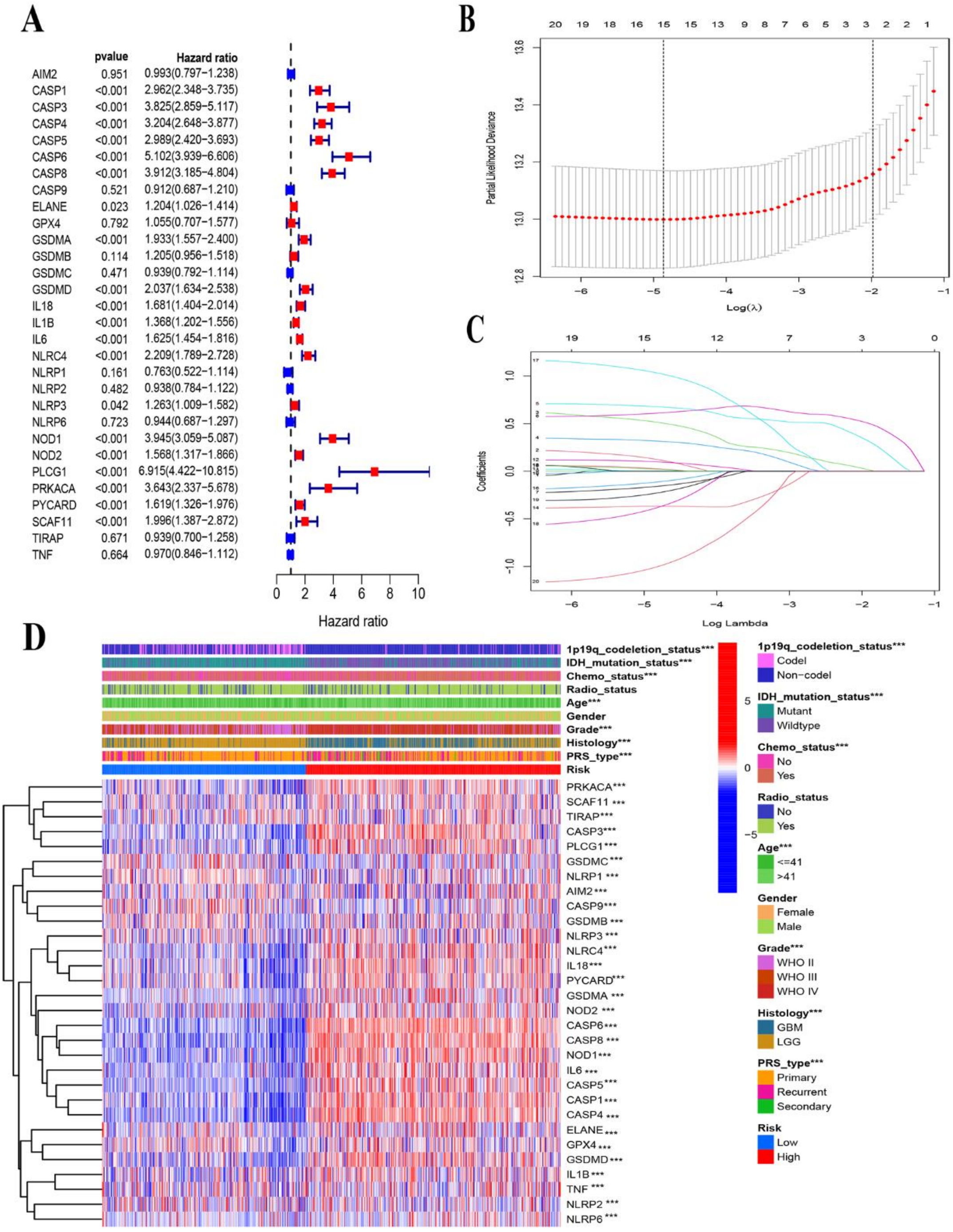
A
pvalue
Hazard ratio
AIM2 0.951 0.993(0.797−1.238)
CASP1 <0.001 2.962(2.348−3.735)
CASP3 <0.001 3.825(2.859−5.117)
CASP4 <0.001 3.204(2.648−3.877)
CASP5 <0.001 2.989(2.420−3.693)
CASP6 <0.001 5.102(3.939−6.606)
CASP8 <0.001 3.912(3.185−4.804)
CASP9 0.521 0.912(0.687−1.210)
ELANE 0.023 1.204(1.026−1.414)
GPX4 0.792 1.055(0.707−1.577)
GSDMA <0.001 1.933(1.557−2.400)
GSDMB 0.114 1.205(0.956−1.518)
GSDMC 0.471 0.939(0.792−1.114)
GSDMD <0.001 2.037(1.634−2.538)
IL18 <0.001 1.681(1.404−2.014)
IL1B <0.001 1.368(1.202−1.556)
IL6 <0.001 1.625(1.454−1.816)
NLRC4 <0.001 2.209(1.789−2.728)
NLRP1 0.161 0.763(0.522−1.114)
NLRP2 0.482 0.938(0.784−1.122)
NLRP3 0.042 1.263(1.009−1.582)
NLRP6 0.723 0.944(0.687−1.297)
NOD1 <0.001 3.945(3.059−5.087)
NOD2 <0.001 1.568(1.317−1.866)
PLCG1 <0.001 6.915(4.422−10.815)
PRKACA <0.001 3.643(2.337−5.678)
PYCARD <0.001 1.619(1.326−1.976)
SCAF11 <0.001 1.996(1.387−2.872)
TIRAP 0.671 0.939(0.700−1.258)
TNF 0.664 0.970(0.846−1.112)
Hazard ratio
B
Partial Likelihood Deviance
Log(λ)
C
Coefficients
Log Lambda
D
1p19q_codeletion_status***
IDH_mutation_status***
Chemo_status***
Radio_status
Age***
Gender
Grade***
Histology***
PRS_type***
Risk
PRKACA***
SCAF11 ***
TIRAP***
CASP3***
PLCG1***
GSDMC***
NLRP1 ***
AIM2***
CASP9***
GSDMB ***
NLRP3 ***
NLRC4***
IL18***
PYCARD***
GSDMA ***
NOD2 ***
CASP6***
CASP8 ***
NOD1***
IL6 ***
CASP5***
CASP1***
CASP4 ***
ELANE***
GPX4 ***
GSDMD***
IL1B***
TNF ***
NLRP2 ***
NLRP6 ***
Codel
Non-codel
Mutant
Wildtype
No
Yes
No
Yes
<=41
>41
Female
Male
WHO II
WHO III
WHO IV
GBM
LGG
Primary
Recurrent
Secondary
Low
High

Figure S5

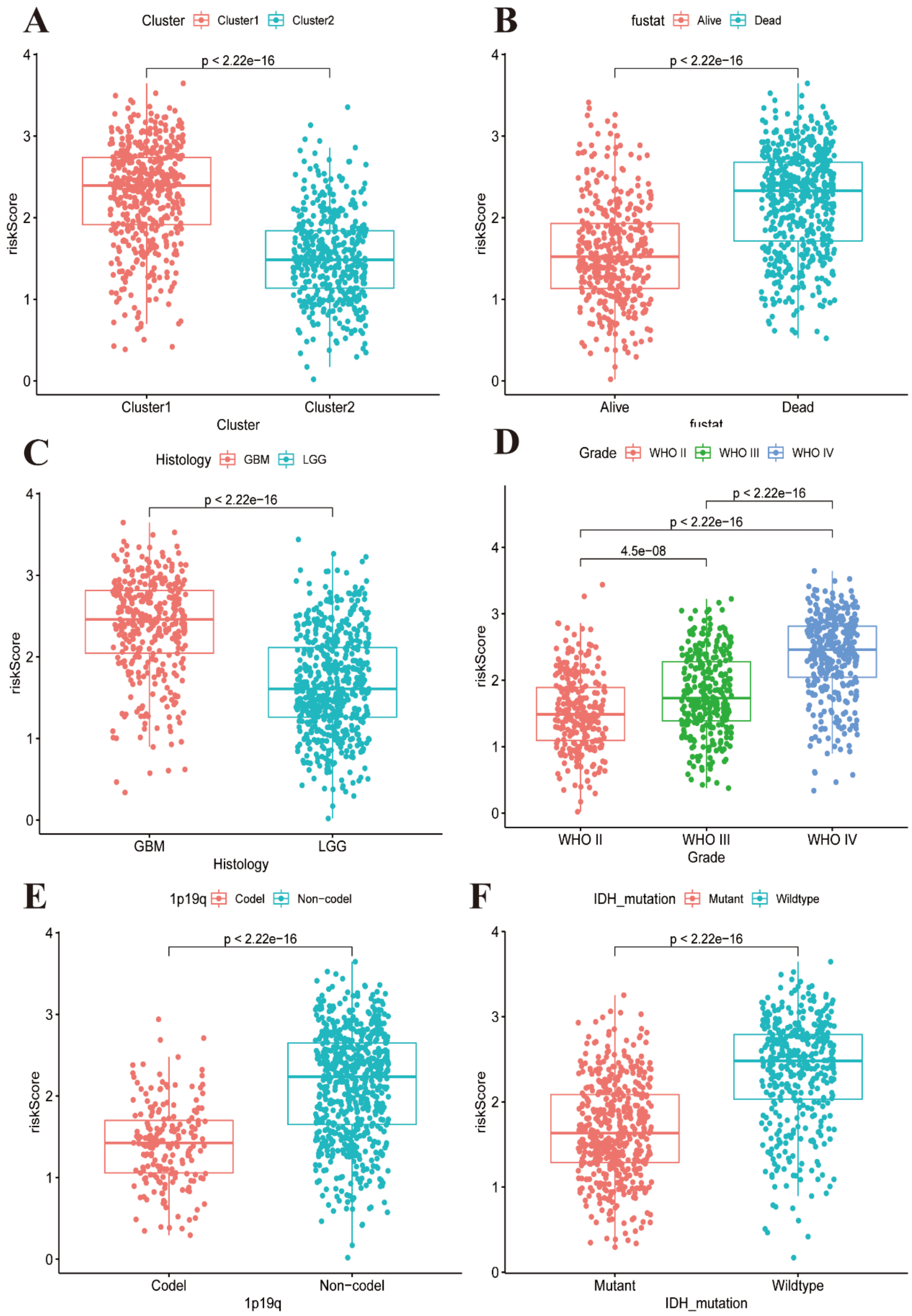
A
Cluster Cluster1 Cluster2
p < 2.22e−16
riskScore
Cluster1
Cluster2
Cluster
B
fustat Alive Dead
p < 2.22e−16
riskScore
Alive
Dead
fustat
C
Histology GBM LGG
p < 2.22e−16
riskScore
GBM
LGG
Histology
D
Grade WHO II WHO III WHO IV
p < 2.22e−16
p < 2.22e−16
4.5e−08
riskScore
WHO II
WHO III
WHO IV
Grade
E
1p19q Codel Non−codel
p < 2.22e−16
riskScore
Codel
Non−codel
1p19q
F
IDH_mutation Mutant Wildtype
p < 2.22e−16
riskScore
Mutant
Wildtype
IDH_mutation

Figure S6

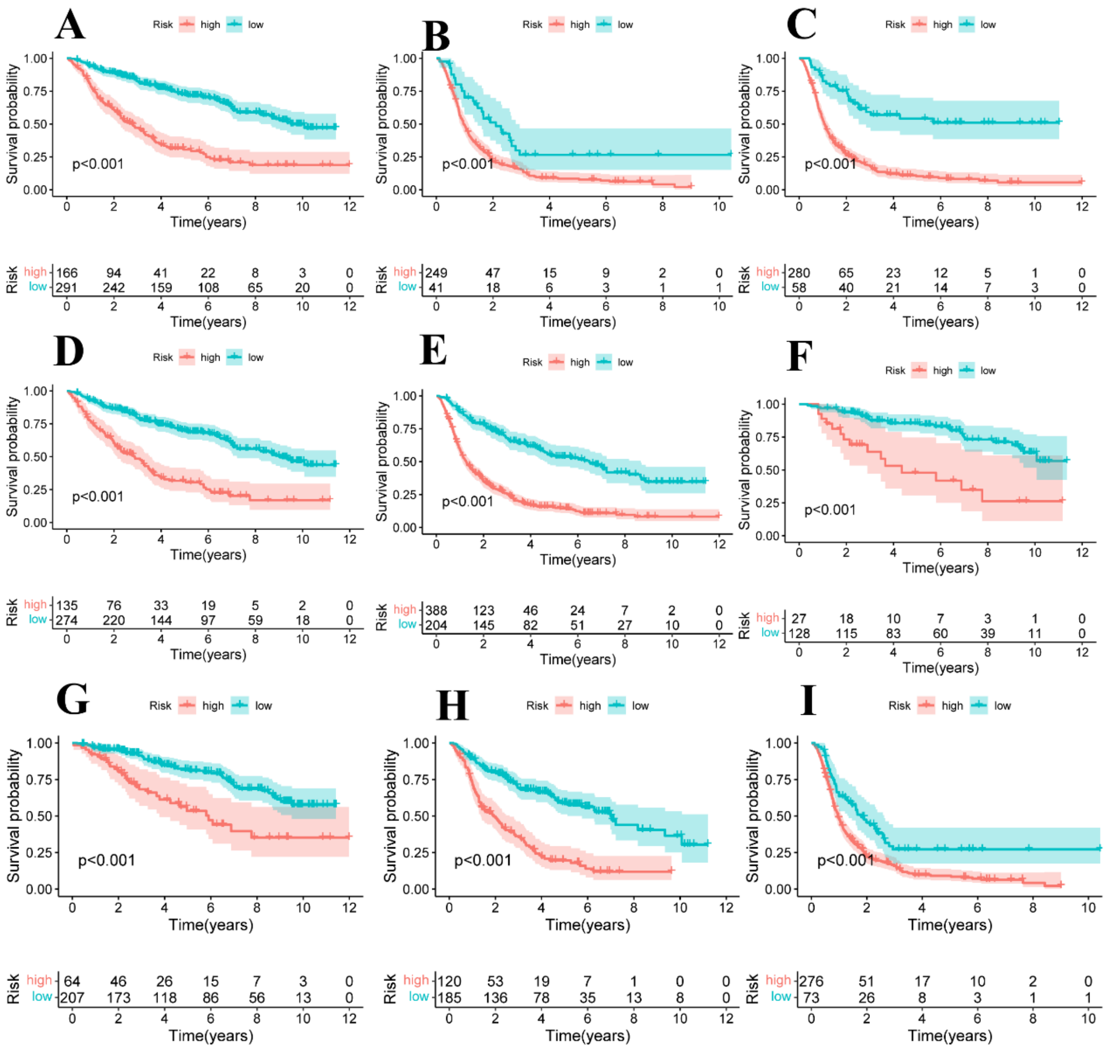
A
Risk high low
Survival probability
p<0.001
Time(years)
Risk high 166 94 41 22 8 3 0
low 291 242 159 108 65 20 0
B
Risk high low
Survival probability
p<0.001
Time(years)
Risk high 249 47 15 9 2 0
low 41 18 6 3 1 1
C
Risk high low
Survival probability
p<0.001
Time(years)
Risk high 280 65 23 12 5 1 0
low 58 40 21 14 7 3 0
D
Risk high low
Survival probability
p<0.001
Time(years)
Risk high 135 76 33 19 5 2 0
low 274 220 144 97 59 18 0
E
Risk high low
Survival probability
p<0.001
Time(years)
Risk high 388 123 46 24 7 2 0
low 204 145 82 51 27 10 0
F
Risk high low
Survival probability
p<0.001
Time(years)
Risk high 27 18 10 7 3 1 0
low 128 115 83 60 39 11 0
G
Risk high low
Survival probability
p<0.001
Time(years)
Risk high 64 46 26 15 7 3 0
low 207 173 118 86 56 13 0
H
Risk high low
Survival probability
p<0.001
Time(years)
Risk high 120 53 19 7 1 0 0
low 185 136 78 35 13 8 0
I
Risk high low
Survival probability
p<0.001
Time(years)
Risk high 276 51 17 10 2 0
low 73 26 8 3 1 1

Figure S7

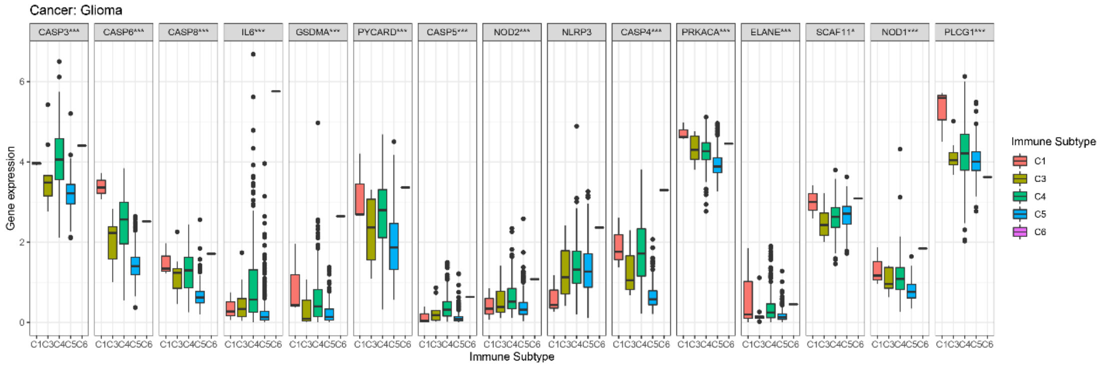

Figure S8

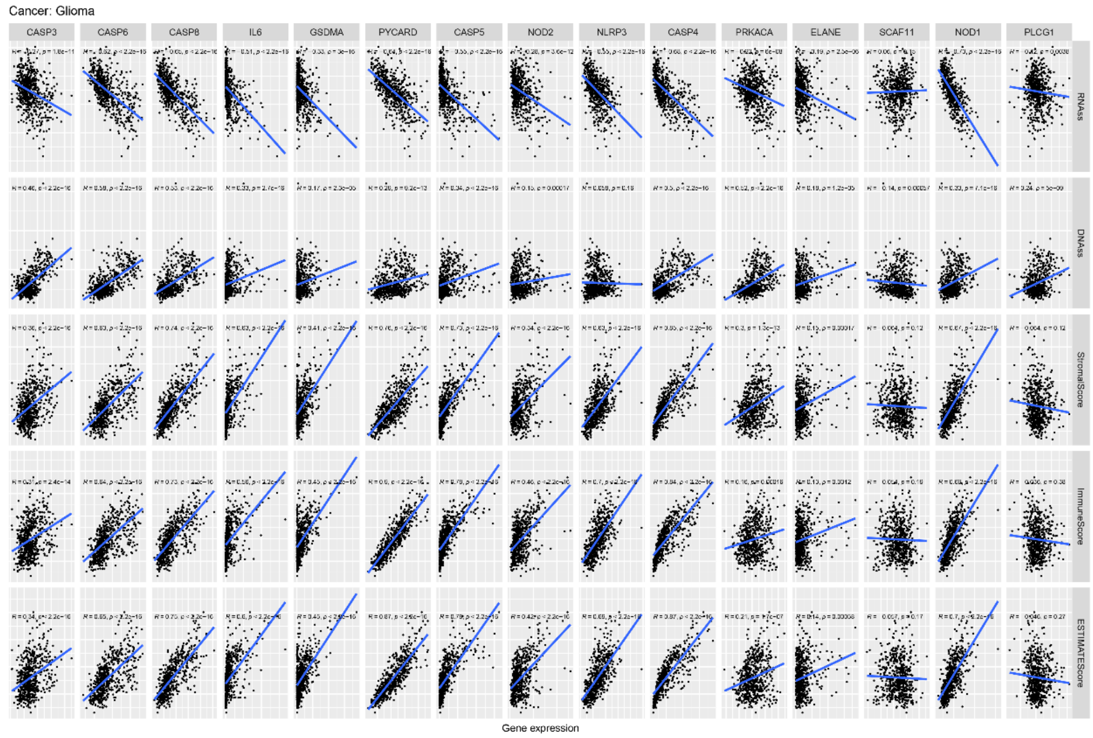

Figure S9

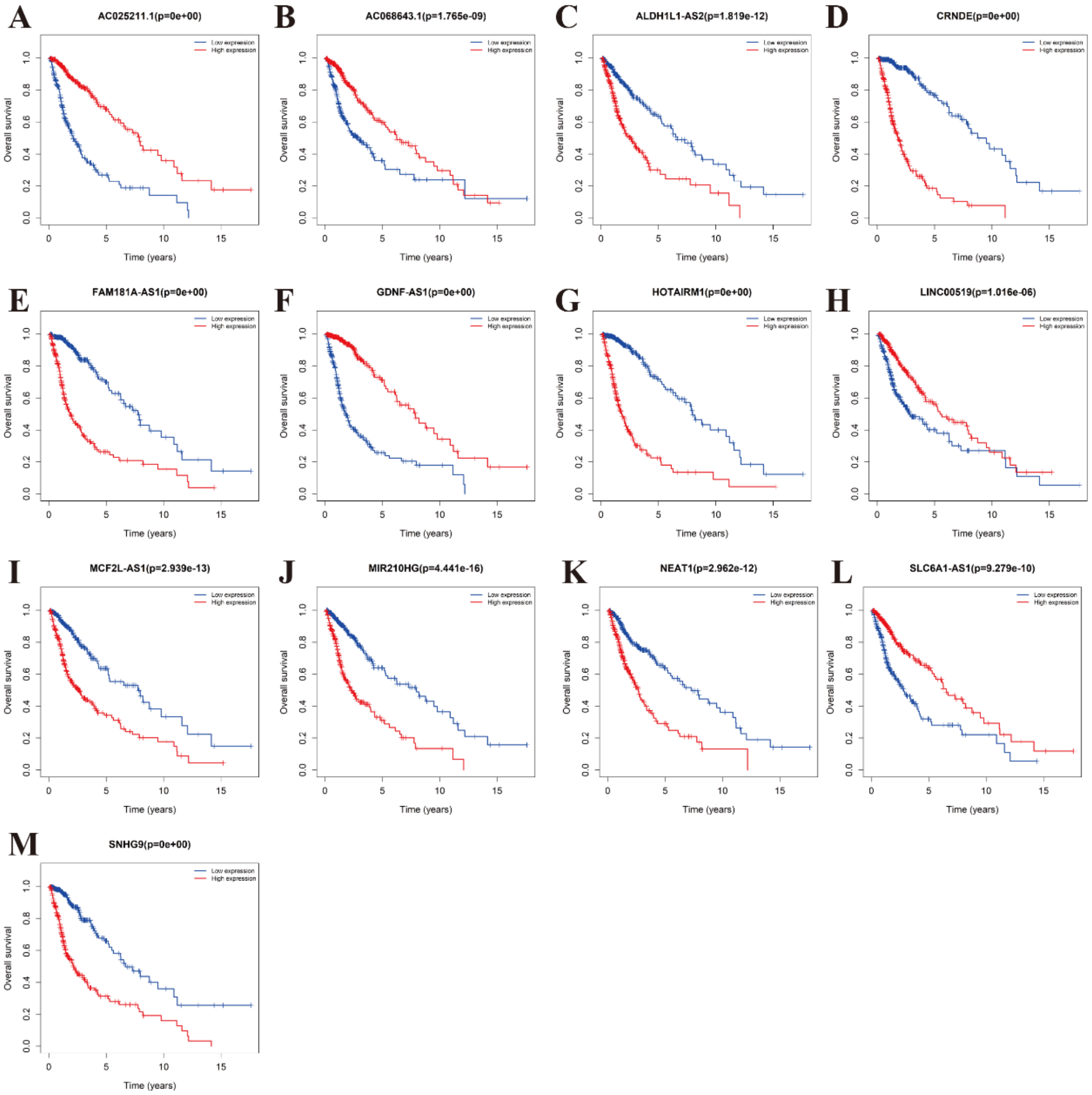
A
AC025211.1(p=0e+00)
B
AC068643.1(p=1.765e-09)
C
ALDH1L1-AS2(p=1.819e-12)
D
CRNDE(p=0e+00)
E
FAM181A-AS1(p=0e+00)
F
GDNF-AS1(p=0e+00)
G
HOTAIRM1(p=0e+00)
H
LINC00519(p=1.016e-06)
I
MCF2L-AS1(p=2.939e-13)
J
MIR210HG(p=4.441e-16)
K
NEAT1(p=2.962e-12)
L
SLC6A1-AS1(p=9.279e-10)
M
SNHG9(p=0e+00)
Low expression
High expression
Overall survival
Time (years)

Figure S10

	pvalue	Hazard ratio
EGFR	<0.001	1.248(1.145−1.361)
EZH2	<0.001	2.056(1.774−2.383)
LZTS1	<0.001	1.615(1.443−1.808)
PRKCE	<0.001	0.290(0.228−0.370)
PTPRD	<0.001	0.473(0.406−0.550)
ELAVL2	<0.001	0.499(0.426−0.585)
FAM46A	<0.001	2.689(2.343−3.086)
SPI1	<0.001	1.719(1.502−1.968)
WASF3	<0.001	0.454(0.396−0.520)
LYPLA1	<0.001	2.396(2.113−2.716)
WEE1	<0.001	2.852(2.486−3.272)
TRIP13	<0.001	2.792(2.371−3.287)
MAP2K3	<0.001	3.900(3.177−4.787)
CPEB3	<0.001	0.210(0.165−0.267)
LATS2	<0.001	3.717(3.057−4.519)
STK40	<0.001	2.653(2.238−3.146)
SPRY2	<0.001	2.376(2.063−2.738)
IKBIP	<0.001	4.118(3.426−4.951)
SHCBP1	<0.001	3.126(2.635−3.709)
SOD2	<0.001	1.666(1.535−1.809)
CAMKK1	0.004	0.809(0.701−0.934)
CYP1B1	<0.001	1.423(1.247−1.622)
BCL7A	<0.001	0.385(0.332−0.445)
CALD1	<0.001	2.708(2.325−3.153)
KPNA2	<0.001	2.940(2.500−3.457)
RHOC	<0.001	2.219(1.903−2.586)
SATB1	<0.001	0.371(0.318−0.432)
LTF	<0.001	1.289(1.239−1.342)
MEIS1	<0.001	2.496(2.085−2.988)
KIF13A	<0.001	0.272(0.224−0.330)
TUB	<0.001	0.401(0.351−0.458)
DPY19L1	<0.001	2.245(1.991−2.531)
JAG1	<0.001	2.015(1.763−2.303)
CAPN5	<0.001	1.746(1.554−1.962)
TMEM100	<0.001	0.631(0.583−0.683)
CADM2	<0.001	0.512(0.460−0.569)
TGFBI	<0.001	1.557(1.436−1.688)
EPHA2	<0.001	2.063(1.806−2.357)
NAMPT	<0.001	1.890(1.734−2.061)
LAMC1	<0.001	2.322(2.055−2.625)

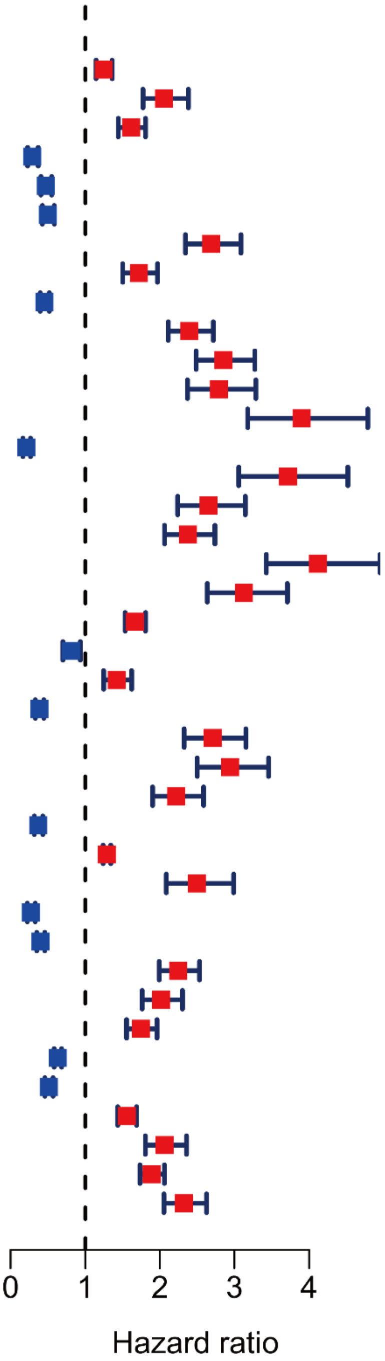

Figure S11

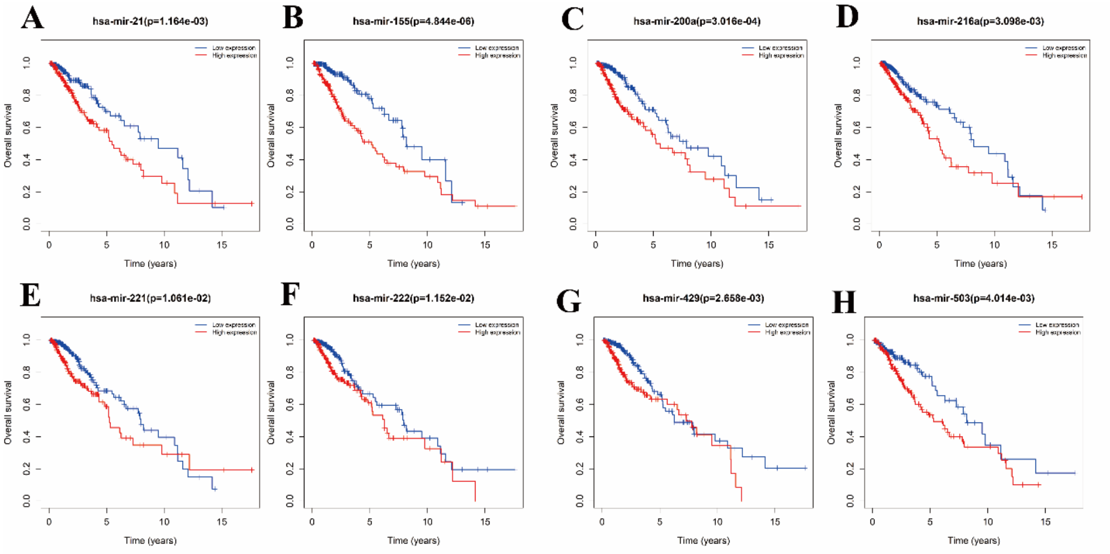
A
hsa-mir-21(p=1.164e-03)
B
hsa-mir-155(p=4.844e-06)
C
hsa-mir-200a(p=3.016e-04)
D
hsa-mir-216a(p=3.098e-03)
E
hsa-mir-221(p=1.061e-02)
F
hsa-mir-222(p=1.152e-02)
G
hsa-mir-429(p=2.658e-03)
H
hsa-mir-503(p=4.014e-03)
Low expression
High expression
Overall survival
Time (years)

Figure S12

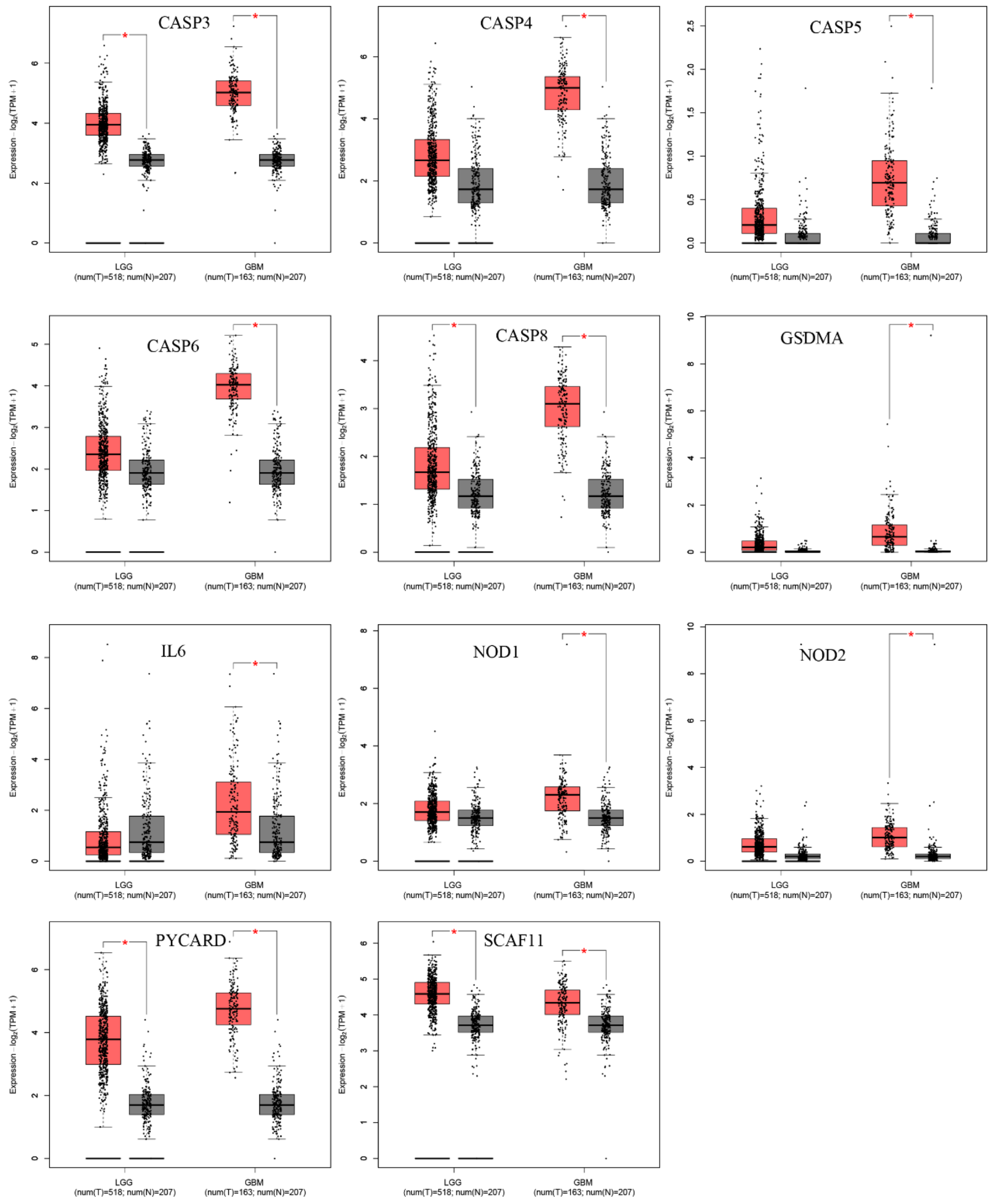

CASP3
CASP4
CASP5
CASP6
CASP8
GSDMA
IL6
NOD1
NOD2
PYCARD
SCAF11
Expression - log2(TPM + 1)
LGG
(num(T)=518; num(N)=207)
GBM
(num(T)=163; num(N)=207)

Figure S13
