# Supplementary material for: Comprehensive Analysis of Pyroptosis-Associated in Molecular Classification, Immunity and Prognostic of Glioma
Source: Front Genet. 2022 Jan 7;12:781538. doi: 10.3389/fgene.2021.781538 (PMC8777075; doi:10.3389/fgene.2021.781538)
Supplement: Supplementary file 7 [file DataSheet2.ZIP › Cell migration assay/Cell migration assay.pdf]

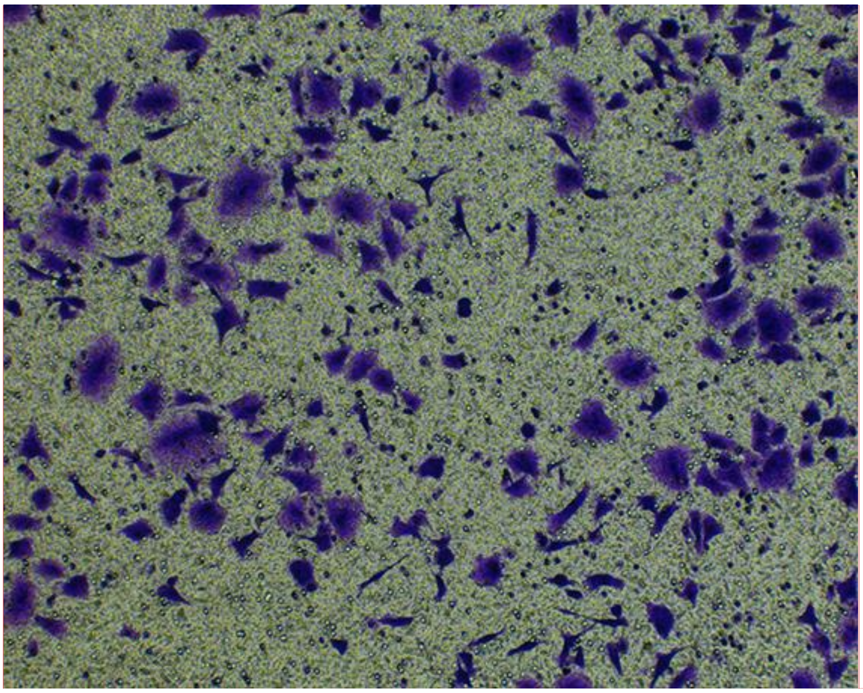

批注 [ZZ1]: LN229-CASP8si-5X

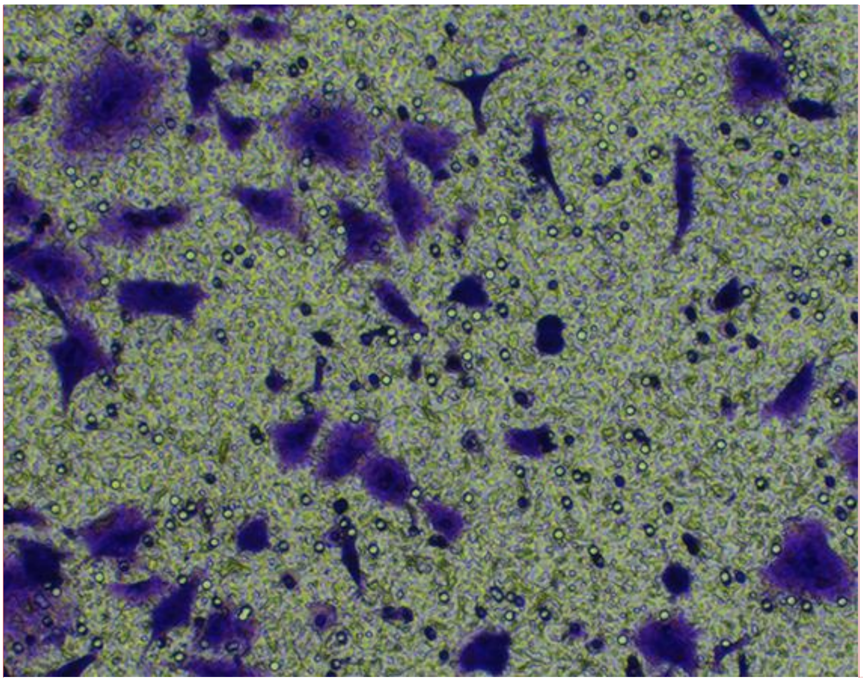

批注 [ZZ2]: LN229-CASP8si-10X

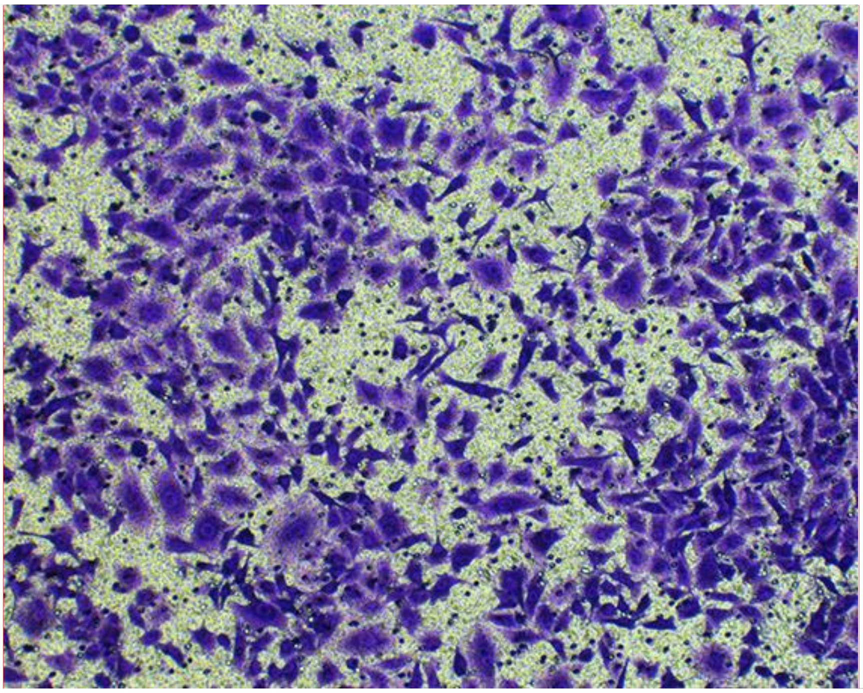

批注 [ZZ3]: LN229-NC-5X

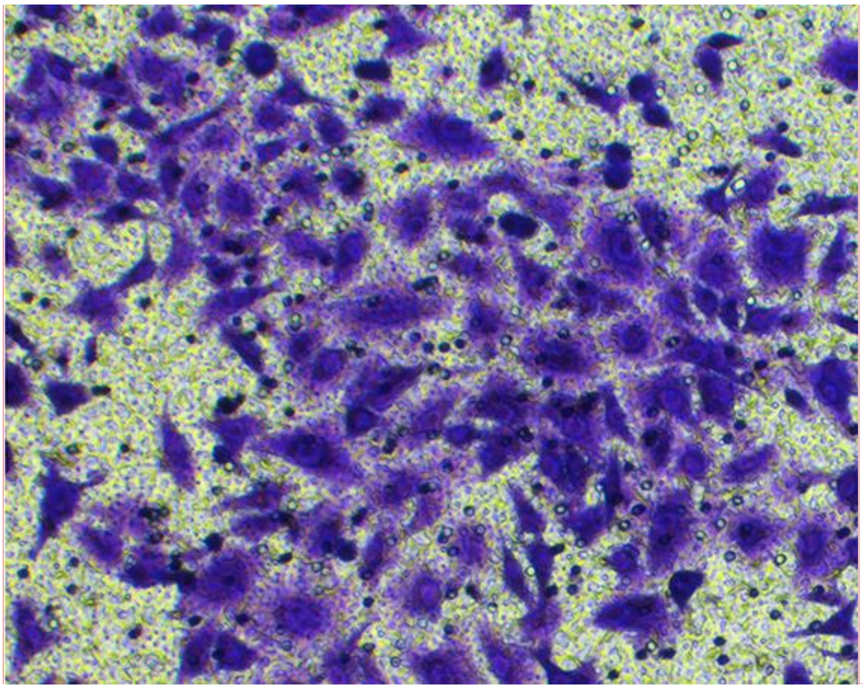

批注 [ZZ4]: LN229-NC-10X

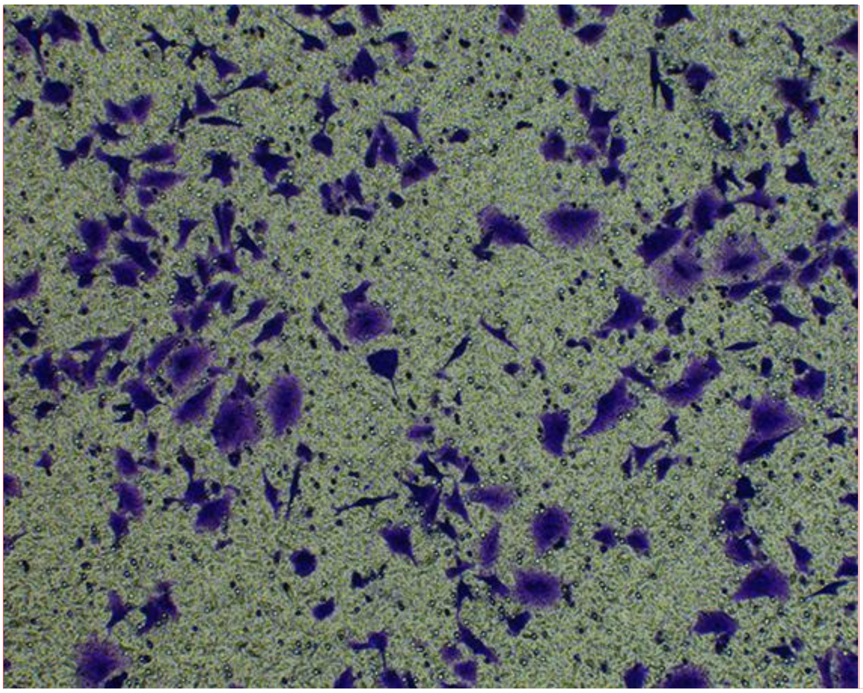

批注 [ZZ5]: U87-CASP8si-5X

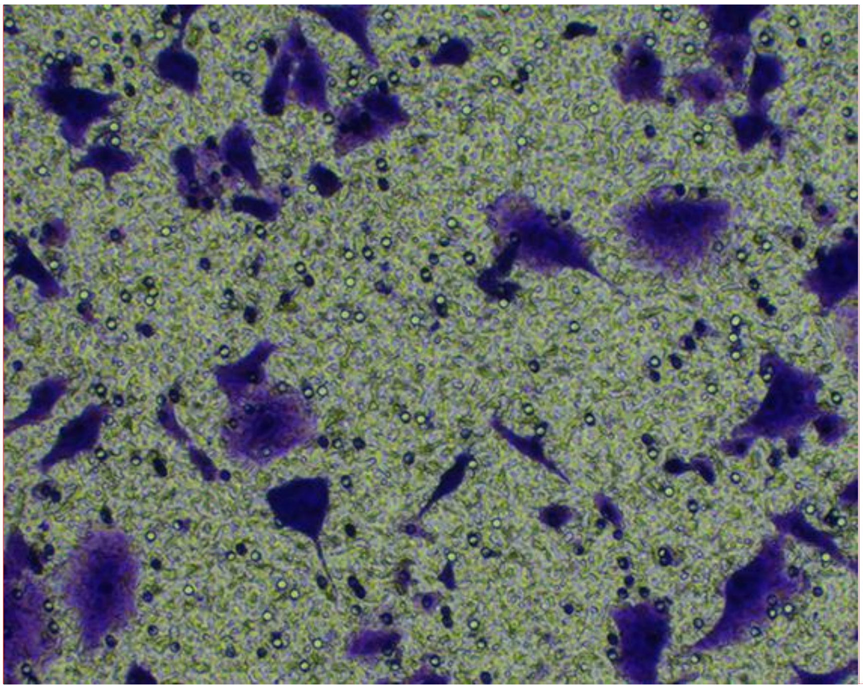

批注 [ZZ6]: U87-CASP8si-10X

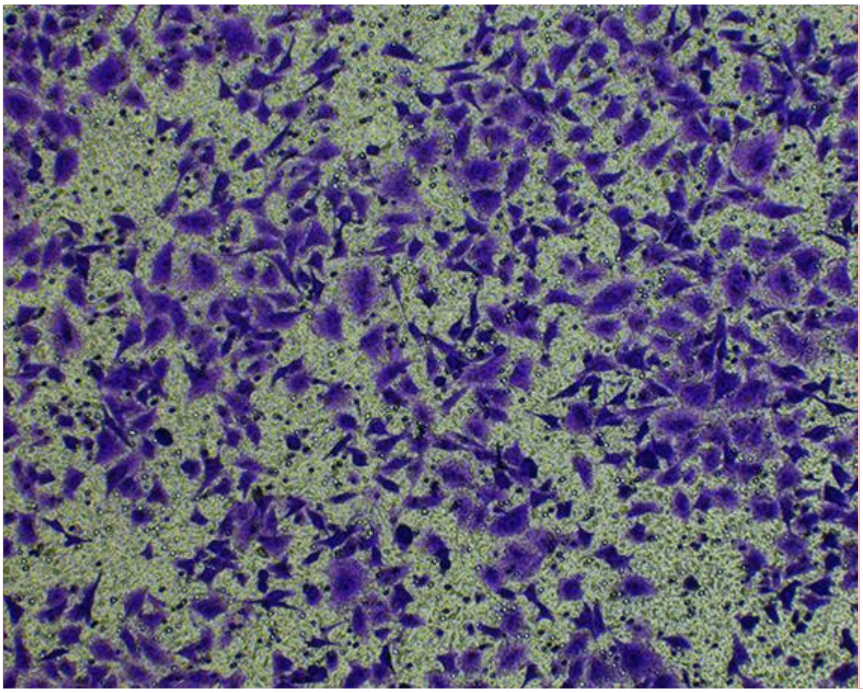

批注 [ZZ7]: U87-NC-5X

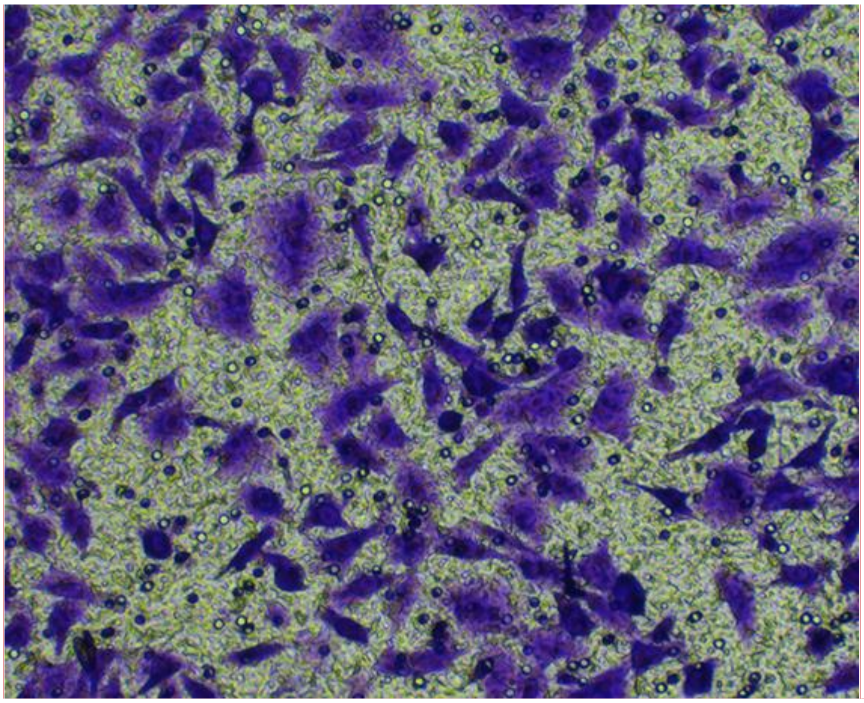

批注 [ZZ8]: U87-NC-10X
